# Early Administration of N-Acetylcysteine Provides Renal and Cardiac Mitochondrial and Redox Protection, Preventing the Development of Cardio-Renal Syndrome Type IV Induced by 5/6NX

**DOI:** 10.3390/antiox14101241

**Published:** 2025-10-16

**Authors:** Karen Peralta-Buendía, Belén Cuevas-López, Fernando E. García-Arroyo, Miriam Díaz-Rojas, Juan Carlos León-Contreras, Alejandro Silva-Palacios, Guillermo Gonzaga, Edilia Tapia, Emma Saavedra, Rogelio Hernández-Pando, José Pedraza-Chaverri, Laura Gabriela Sánchez-Lozada, Omar Emiliano Aparicio-Trejo

**Affiliations:** 1 Department of Cardio-Renal Physiopathology, National Institute of Cardiology Ignacio Chávez, Mexico City 14080, Mexico; 317061851@quimica.unam.mx (K.P.-B.); bcuevasl1400@alumno.ipn.mx (B.C.-L.); enrique.garcia@cardiologia.org.mx (F.E.G.-A.); jose.gonzaga@cardiologia.org.mx (G.G.); edilia.tapia@cardiologia.org.mx (E.T.); laura.sanchez@cardiologia.org.mx (L.G.S.-L.); 2 Division of Medicinal Chemistry and Phytochemistry, College of Pharmacy, The Ohio State University, Columbus, OH 43210, USA; diaz.530@osu.edu; 3 Experimental Pathology Section, National Institute of Medical Sciences and Nutrition “Salvador Zubirán”, Mexico City 14000, Mexico; carlos.leonc@incmnsz.mx (J.C.L.-C.); rogelio.hernandezp@incmnsz.mx (R.H.-P.); 4 Department of Cardiovascular Biomedicine, National Institute of Cardiology Ignacio Chávez, Mexico City 14080, Mexico; alejandro.silva@cardiologia.org.mx; 5 Departamento de Bioquímica, Instituto Nacional de Cardiologia Ignacio Chávez, Mexico City 14080, Mexico; emma.saavedra@cardiologia.org.mx; 6 Laboratorio F-315, Departamento de Biología, Facultad de Química, Universidad Nacional Autónoma de México, Mexico City 04510, Mexico; pedraza@unam.mx

**Keywords:** cardio-renal syndrome type 4, nephrectomy, N-acetylcysteine, mitochondria bioenergetics, mitochondrial redox regulation, mitochondrial biogenesis

## Abstract

Chronic kidney disease (CKD) cardiac impairment is manifested as cardio-renal syndrome type 4 (CRS-IV). The kidneys and heart are highly dependent on mitochondria; thus, bioenergetics and redox and biogenesis alterations are critical in CKD and heart damage. Most previous studies have focused on the advanced stage of CRS-IV, but mitochondrial impairment onset in the early stages and its pathological pathways are poorly understood. In this work, we characterized mitochondrial bioenergetics, biogenesis and redox impairment in both tissues in the early stages after CKD and analyzed their relationship with CRS-IV in a CKD model with 5/6 nephrectomy (NX). We found the first cardiac mitochondrial alterations 10 days after surgery, together with an increase in plasma cardio-renal connectors, derived from renal mitochondrial damage. Oxidative phosphorylation capacity decreased and uncoupling led to oxidative stress, inflammation, cardiac hypertrophy and ejection fraction reduction, triggering CRS-IV. N-acetylcysteine (NAC) administration prevented mitochondrial alterations in both organs and heart damage. Interestingly, the protective effects of NAC correlated with SIRT1/3-PGC-1α pathway overactivation. These results suggest that mitochondrial biogenesis induction and redox regulation protection in the early stages after renal damage serve as a strategy to prevent bioenergetic alterations in the kidneys and heart, preventing inflammation and CRS-IV development.

## 1. Introduction

The heart and kidneys maintain a feedback interaction essential for overall homeostasis. Therefore, alterations in this interaction favor the development of several pathologies known as cardio-renal syndrome (CRS) [1]. In this sense, CRS type 4 (CRS-IV) is established when chronic kidney disease (CKD) results in cardiovascular abnormalities, which occurs in approximately 50% of patients [2,3,4,5]. As a consequence, heart failure represents the principal cause of mortality in CKD patients [6,7], making this pathology a growing worldwide health problem.

Mitochondria are currently considered key regulators in cell signaling and metabolic processes, transcending their function for energy production [8]. Our previous studies in CRS models demonstrated that mitochondria and cardiac apoptosis are crucial in cardiac dysfunction development [9,10]. A primary organelle involved in cellular reactive oxygen species (ROS) balance, mitochondria act as a redox center in metabolic regulation, and protein turnover is tightly coordinated with mitochondrial redox balance [11]. Therefore, proteins and regulatory factors of mitochondrial biogenesis and protein turnover, OXPHOS, β-oxidation, Krebs cycle and sirtuins (SIRT), are sensitive to changes in mitochondrial redox balance [12]. Thus, disruption of the mitochondrial glutathione /glutathione disulfide ratio (GSH/GSSG) and NADP^+^/NADPH ratio plays a key role in CKD progression; therefore, interventions that normalize this balance are studied as potential treatments for protecting mitochondria in the heart and kidneys [13]. In particular, metabolic reprogramming and mitochondrial biogenesis via the sirtuin 1/3 (SIRT1/3)-peroxisome proliferator-activated receptor gamma coactivator 1-alpha (PGC-1α) pathway must be well researched [1]. On the other hand, different studies in advanced stages of CKD and in uremic cardiomyopathy have shown that uremia and uremic toxins like indoxyl sulfate (IS) induce heart mitochondrial respiration impairment, inflammation, oxidative stress and cardiomyocytes apoptosis [14,15,16]. This mitochondrial impairment is related to the uremic toxins’ capacity to induce SIRT1/3-PGC-1α axis downregulation [17,18,19,20]. However, it must be highlighted that in CKD models like 5/6 nephrectomy NX5/6 in the early stage of evolution, during which the uremic toxins have not increased significantly in blood, renal mitochondrial respiratory impairment and PGC-1α downregulation have been reported [21,22,23]. Likewise, our previous studies suggested that in short-term experimental end-points, cardiac mitochondrial and redox alteration, may trigger mitochondrial biogenesis reduction and CRS development [9,10]. However, in the early stage of CKD evolution, the molecular connections between SIRT1/3-PGC-1α dysregulation, redox imbalance and inflammatory alterations in the heart and kidneys in CRS-IV have not been deeply explored [1].

N-acetylcysteine (NAC) administration has been reported to replenish intracellular redox metabolites [24], preventing redox imbalance and mitochondrial impairment in renal, cardiac and cardio-renal diseases [13,25,26]. However, current understanding of NAC’s role in modulating mitochondrial redox balance, mainly its role in SIRT1/3 modulation, in the context of CRS-4 is still poor. Thus, this work aims to determine the role of cardiac mitochondrial bioenergetics and SIRT1/3-PGC-1α pathway alterations in renal and heart tissues during CRS-IV development in the early stages in the NX5/6 rat model. In addition, we aim to determine whether the possible prevention of these alterations by NAC is related to its capacity to regulate redox signaling and restore mitochondrial biogenesis in both tissues.

## 2. Materials and Methods

### 2.1. Reagents

Reagents adenosine 5′-diphosphate sodium (ADP), antimycin A from Streptomyces, bovine serum albumin (BSA) free of fatty acids, β-mercaptoethanol, bromophenol blue sodium salt, carbonyl cyanide m-chlorophenylhydrazone (CCCP), 1-Chloro-2,4-dinitrobenzene (CDNB), cytochrome C, 2,3-Dimethoxy-5-methyl-6-decyl-1,4-benzoquinone/decylubiquinone (DUB), 2,6-dichlorophenolindophenol (DCPIP), 5,5′-dithio-bis-(2-nitrobenzoic) acid (DTNB), DL-ditiotreitol solution (DTT), ethylene-bis(oxyethylenenitrilo)tetraacetic acid (EGTA), glucose-6-phosphate Dehydrogenase from baker’s yeast (G6PDH), glutathione peroxidase (GPx), reduced glutathione (GSH), glutathione disulfide (GSSG), glutathione reductase (GR), glutamic acid, glutaraldehyde, hexokinase, 4-(2-hydroxyethyl)-1-piperazineethanesulfonic acid (HEPES), peroxidase from horseradish lyophilized powder (HRP), malic acid, manganese (II) chloride (MgCl_2_), mannitol, NAC, β-Nicotinamide adenine dinucleotide phosphate reduced (NADPH) and oxidized (NADP^+^), β-Nicotinamide adenine dinucleotide reduced (NADH) and oxidized (NAD^+^), nitro blue tetrazolium (NBT), paraformaldehyde, potassium cyanide (KCN), potassium lactobionate, polyethylene glycol sorbitan monolaurate (Tween-20), rotenone, safranin O, sodium azide, sodium deoxycholate, sodium dodecyl sulfate (SDS), disodium hydrogen phosphate (Na_2_HPO_4_), monosodium phosphate (NaH_2_PO_4_), sodium glutamate, sodium chloride (NaCl), sodium fluoride (NaF), sodium succinate, sodium orthovanadate (Na_3_VO_4_), sodium malate, sucrose, taurine, tetramethyl-p-phenylenediamine (TMPD), thiamine pyrophosphate, triton X-100, trizma base and hydrochloride (Tris-HCl), and uranyl acetate were acquired from Sigma-Aldrich (St. Louis, MO, USA), as were the NADP^+^/NADPH quantitation and creatine kinase (CK) activity assay kits. Commercial kits for assessing blood urea nitrogen (BUN) and plasma creatinine were acquired from Spinreact (Girona, Spain). The sodium pentobarbital (SedalphorteMR) was acquired from Salud y Bienestar Animal S.A. de C.V. (Mexico City, Mexico). Sodium bicarbonate (NaHCO_3_), H_2_O_2_, ethyl alcohol, ethylenediaminetetraacetic acid disodium salt dihydrate (EDTA), and potassium hydroxide (KOH) were purchased from JT Baker (Xalostoc, Estado de Mexico, Mexico). Non-fat dry milk, beta-actin, brain natriuretic peptide (BNP), interleukin 1 beta (1β), cardiac troponin, nuclear respiratory factor 1 (NRF1), nuclear respiratory factor 2 (NRF2), tumor necrosis factor (TNF)-alpha, peroxisome proliferator-activated receptor gamma coactivator 1-alpha (PGC-1α), peroxisome proliferator-activated receptor gamma coactivator 1-beta (PGC-1β), and 3 (SIRT3) antibodies were acquired from Santa Cruz Biotechnology (Dallas, TX, USA). Adenine nucleotide translocator (ANT) antibody, rat TNF-α and rat IL-1 beta ELISA kits were purchased from Abcam (Cambridge, MA, USA). Voltage dependence anion channel (VDAC) and sirtuin 1 (SIRT1) antibodies were acquired from Cell Signaling (Boston, MA, USA). The OxPhos Rodent WB Antibody Cocktail was purchased from Invitrogen-Thermo Fisher (Waltham, MA, USA). The catalog numbers and solutions used for each antibody are shown in the Supplementary Material Table S1. The protease inhibitor cocktail and phosphoenolpyruvate (PEP) were purchased from Roche Applied Science (Mannheim, Germany).

### 2.2. Experimental Design

All the experimental procedures were approved by the Comité Interno para el Cuidado y Uso de Animales de Laboratorio (CICUAL; the Institutional Animal Care Committee) at the National Institute of Cardiology Ignacio Chávez (protocol number: INC/CICUAL/011/2023) and at the Facultad de Química (protocol number: OFICIO/FQ/CICUAL/545/24). The experimental procedures were also conducted following the Mexican Official Norm Guides for the use and care of laboratory animals (NOM-062-ZOO-1999). Meanwhile biological residue disposition was conducted following NOM-087-SEMARNAT-SSA1-2002. The experimental design consisted of male Wistar rats, divided into 4 groups (initial body weight = 250–300 g), with n = 6 per group: (1) Sham-operated rats; (2) 5/6NX group: five-sixths nephrectomy was performed using the protocol described previously in [21]; (3) NAC+NX: animals were pretreated with NAC (300 mg/kg, intragastrical) 2 h before surgery and the NAC dose was selected based on its protective effect in CRS-IV in a previous study [25]; (4) NAC: animals only received NAC administration. Animals were housed in a special designed room with a controlled temperature and 12–12 h light–dark cycle. Rats had free access to water and food ad libitum. Then, several days after surgery, the animals were anesthetized using sodium pentobarbital (60 mg/kg), and blood was collected from the abdominal aorta. After plasma separation by centrifugation, it was stored at 4 °C; meanwhile, tissues were dissected and stored at −80 °C.

### 2.3. Cardio-Renal Damage Markers and Evaluation of Cardiac Function by Echocardiography

Creatinine and BUN commercial kits from Spinreact (Girona, Spain) were employed to determine the increase in renal damage markers in plasma. The plasma activity of creatine kinase (CK) was also evaluated using commercial kits from Sigma-Aldrich (St. Louis, MO, USA). Uric acid in plasma was measured using a commercial assay kit quantichron™ (DIUA-250) (BioAssay Systems, Hayward, CA, USA). Uric acid was extracted from the heart and renal cortex tissue according to methods previously published, and the concentration was measured and corrected by protein concentration determined using the Lowery method. Meanwhile, inflammatory and heart damage markers, namely BNP, IL-1β, IL-6 and cardiac troponin, in plasma were evaluated by Western blot (WB) using the methodology described below.

Echocardiography: For analysis of heart function and morphology, rats were anesthetized with 5% isoflurane, and 2.5–3% isoflurane was used to maintain their heart rate between 300 and 400 beats per minute. The rats were then placed supine on a temperature-controlled platform for echocardiography using the Prospect T1 high-frequency ultrasound system (Scintica Instrumentation, Inc., Waterloo, ON, Canada) with a 20 MHz transducer (S-Sharp Corporation, New Taipei City, Taiwan). Respiratory and heart rate were monitored with a four-lead electrocardiogram at extremities with electrogel. Body temperature was determined by a rectal probe. A heating pad for rats was employed to maintain it at 37 °C. A razor and depilatory were employed to remove the thoracic hair. Parasternal long-axis B-mode and M-mode images were recorded to visualize left ventricular (LV) anterior and posterior wall motion during diastole. In addition, cardiac output (CO), fractional shortening (FS), end-diastolic volume (EDV), end-systolic volume (ESV), ejection fraction (EF) and stroke volume (SV) were determined according to our previous report [27]. After each echocardiography, the rats were placed in a heated box at 30 °C for recovery until they were fully awake, after which we continued with subsequent evaluations.

### 2.4. Kidney and Heart Histology

Kidneys and hearts were removed instantly after euthanasia; the tissues were cut in half, and fixation was carried out via immersion using a solution of paraformaldehyde/glutaraldehyde (4%/1.5%) at pH = 7.2. Slices of 1 mm in width were cut, dehydrated and embedded in paraffin; 5 μm wide slices were obtained, and Masson trichrome and hematoxylin/eosin (H&E) staining was performed as previously described [25].

### 2.5. Protein Extraction and WB

To extract protein from the kidneys and heart, the tissues were homogenized at 4 °C in radioimmunoprecipitation (RIPA) buffer (2 mM EDTA, 1 mM EGTA, 150 mM NaCl, 5 mM NaF, 1 mM Na_3_VO_4_, 1 mM PMSF, 40 mM Tris-HCl, 0.5% sodium deoxycholate, 0.5% SDS at pH = 7.6) with a protease inhibitor cocktail. Tissues were homogenized in a Potter–Elvehjem homogenizer and then centrifuged for 10 min at 15,000× *g* at 4 °C. The supernatants were then collected. Total protein was quantified by using the Lowry method. Then, the corresponding protein quantities were denatured via boiling for 10 min and diluted 1:6 in Laemmli sample buffer (60 mM Tris-HCl, pH = 6.8, 2% SDS, 10% glycerol, 5% β-mercaptoethanol, 0.01% bromophenol blue). Samples (30 μg kidney or 40 μg heart) and a molecular weight ladder were loaded in SDS-polyacrylamide gels, and then electrophoresis was carried out in the electrophoresis chamber. Polyvinylidene fluoride (PVDF) membranes were employed to transfer the proteins, and non-fat dry milk at 5% in TBS-T at 0.4% was employed to block the membranes at room temperature for 1.5 h. Afterwards, incubation with the appropriate primary antibody was performed overnight at 4 °C, followed by incubation with the fluorescent secondary antibody (dilution 1:10,000–5000 on TBST buffer) at room temperature for 1.5 h in the dark. Band identification was achieved by fluorescence using an Odyssey Sa scanner (LICOR Biosciences, Lincoln, NE, USA). Bands were analyzed with Image Studio Lite software version 5.2 (LICOR Biosciences, Lincoln, NE, USA). Meanwhile, IL-1 β blood levels in plasma were also determined by using ELISA commercial kits acquired from Abcam (Cambridge, MA, USA).

### 2.6. Heart and Kidney Mitochondria Isolation

Heart and kidney tissues were cooled via immersion in PBS at 4 °C after animal sacrifice. Mitochondria from each tissue were isolated by differential centrifugation, as previously described [25,28], in mitochondrial isolation buffer (225 mM D-mannitol, 75 mM sucrose, 1 mM EDTA, 5 mM HEPES, 0.1% BSA, pH = 7.4) and washed in BSA-free mitochondrial isolation buffer. The heart mitochondrial pellet was resuspended in 120 µL and the kidney mitochondrial pellet in 200 µL of BSA-free isolation buffer. Finally, the Lowry method was employed to determine the total protein concentration [28].

### 2.7. Mitochondrial Respiratory Parameters and Membrane Potential (ΔΨm)

Respiratory state evaluation was performed with fresh isolated kidney and heart mitochondria using a high-resolution respirometer (Oxygraph O2k, OROBOROS, Innsbruck, Austria) at 37 °C. As previously described [25,28], renal (500 µg) and heart (500 µg) mitochondria were run in the chamber with 2 mL of MiR05 buffer (0.5 mM EGTA, 3 mM MgCl_2_, 60 mM K-lactobionate, 20 mM taurine, 10 mM KH_2_PO_4_, 20 mM HEPES, 110 mM sucrose, and 1 g/L essentially FA-free BSA, pH = 7.4). The experiment began with mitochondrial addition, followed by the addition of complex I plus complex II (CI + CII)-linked substrates (5 mM pyruvate, 2 mM malate, 10 mM glutamate plus 10 mM succinate) [22,28]. In addition, 2.5 mM ADP was added to induce respiration in state 3 (S3); meanwhile, respiration in state 4o (S4o) was induced by oligomycin 2.5 μM. Residual respiration (ROX) was induced by 1 µM rotenone plus 2.5 µM antimycin A and was used to correct all respiratory parameters. The respiratory control ratio (RC) was defined as the S3/S4o ratio, and OXPHOS-associated respiration (P) was defined as S3-S4o [26,29]. Total protein content was used to normalize respiratory parameters.

ΔΨm was evaluated in the previously described respiratory states by determining the changes in 5 µM safranin O fluorescence, as previously described [26]. CI + CII substrates were added after mitochondria addition to stimulate state 2 (S2), and 2.5 mM ADP was also employed to induce S3; meanwhile, 2.5 μM oligomycin was used to induce S4o. To correct safranine non-specific interactions, CCCP was used to fully dissipate the potential. The changes in ΔΨm were reported as the changes in the measurable concentration of safranin O (ΔμM of S) in each state compared to the full-potential dissipation state and normalized per milligram of protein (ΔμM/mg).

### 2.8. Mitochondrial H_2_O_2_ Production Rates

Mitochondrial H_2_O_2_ rates at 37 °C were evaluated in freshly isolated mitochondria of renal and heart tissues, as previously described [25,26], using O2k-Fluorometer (OROBOROS, Innsbruck, Austria) with Amplex red as a probe. Briefly, renal or heart mitochondria were resuspended in MiR05 plus 0.5 U/mL HRP. Calibration curves were employed to ensure the linearity of the assay, as previously described [25,26].

### 2.9. Activity of Mitochondrial Respiratory Complexes and ATP Synthase, and NADP^+^/NADPH Ratio

The activity of the mitochondrial complexes was evaluated as previously described in [28]. The CI activity evaluation was based on the oxidation of NADH while reducing DUb to DUbH_2_, after which DUbH_2_ is then oxidized by DCPIP. Therefore, the reduction in DCPIP absorbance at 600 nm can be used to evaluate CI activity. The evaluation of CII activity used a similar system in which succinate was employed as a substrate in addition to 2.5 µM rotenone. CII reduces Dub and the reduction in DCPIP absorbance at 600 nm can be used to evaluate CII activity. The activity of complex III (CIII) was evaluated using cytochrome *c* reduction, which is followed by an absorbance increase at 550 nm after adding DUbH_2_. Complex IV (CIV) activity was evaluated according to the decrease in the absorbance at 550 nm due to the oxidation of cytochrome c by complex IV; 1 mM sodium azide was used as an inhibitor of CIV. To evaluate ATP synthase activity, a hexokinase (HK)-glucose 6 phosphate dehydrogenase (G6PDH) enzyme-linked assay was employed, in which the reduction of NADP^+^ at 340 nm is proportional to ATP synthase activity [21]. Oligomycin (20 mM) was used as a specific inhibitor [21]. Absorbance measurements were performed at 37 °C using a Cytation 7 microplate reader (Biotek Instruments, Winooski, VT, USA). The specific activity of each complex was determined by subtracting the activity in the presence of the appropriate inhibitor from the non-inhibited activity, and the results were expressed as nmol per minute per milligram of protein (nmol/min/mg).

The mitochondrial NADP^+^/NADPH ratio was determined in fresh isolated renal or heart mitochondria using a commercial kit from Sigma-Aldrich (St. Louis, MO, USA). Absorbance measurements were performed at 37 °C using a Cytation 7 microplate reader (Biotek Instruments, Winooski, VT, USA).

### 2.10. Evaluation of Antioxidant Enzyme Activities

Mitochondrial, heart or kidney homogenates were used to determine antioxidant enzyme activities, as previously described [25,26]. In brief, superoxide dismutase (SOD) activity was evaluated according to the interference in the oxidation of the NBT probe at 560 nm. Glutathione S-transferase (GST) activity was evaluated according to the increase in the absorbance at 340 nm due to GSH-CDNB formation. Glutathione reductase (GR) activity was determined according to the disappearance of NADPH absorbance at 340 nm. Glutathione peroxidase (GPx) activity was determined according to the disappearance of NADPH at 340 nm in a coupled reaction with GR. Catalase activity was determined according to H_2_O_2_ decomposition at 240 nm using the Aebi method. All measurements were performed at 37 °C using a Cytation 7 microplate reader (Biotek Instruments, Winooski, VT, USA).

### 2.11. In Silico NAC Interaction with PPAR-α and Nuclear Respiratory Factor (NRF2)

To evaluate the potential NAC activity on mitochondrial biogenesis factors, in silico unspecific docking was performed using SwissDock version 2, after having used Autodock Tools version 1.5.6 [30] and AutoDock Vina version 1.2.0 [31] for a more precise interaction, with a grid of 60 × 60 × 60 Å. Previously reported positive control interactions were used for coordinate selection. Chimera software version 1.17.3 was used for protein and figure preparation; finally, Spartan (Wavefunction Inc., Irvine, CA, USA) was used for the optimization of the antagonist compound, considering the protonation form for each case. The crystallographic structures were downloaded from the Protein Data Bank; for molecular docking, we used 6KB4 in the case of PPAR-α and 1AWC for NRF2, and in each case an appropriate positive control was selected.

### 2.12. Electron Microscopy

Small myocardium small fragments were fixed on 2.5% glutaraldehyde in 0.15 M cacodylate buffer. Then, 1% osmium tetroxide was employed post fixing with ascending concentrations of ethyl alcohol to achieve dehydration. The fragments were infiltrated in epoxy resin. Uranyl acetate and lead citrate were used for contrast, and grids were observed with an electron microscope (Tecnai Spirit BioTwin, FEI, Hillsboro, OR, USA).

### 2.13. Statistics

Data are presented as the mean ± the standard error of the mean (SEM). Comparisons among groups were tested using ANOVA followed by Tukey’s post hoc test or the Kruskal–Wallis test followed by Dunn’s post hoc analysis (for WB analysis). Significant differences were considered when the *p* value was <0.05 using Graph-Pad Prism 6 (San Diego, CA, USA).

## 3. Results

### 3.1. NAC Prevents the Increase of Cardiorenal Syndrome

**Figure 1:**
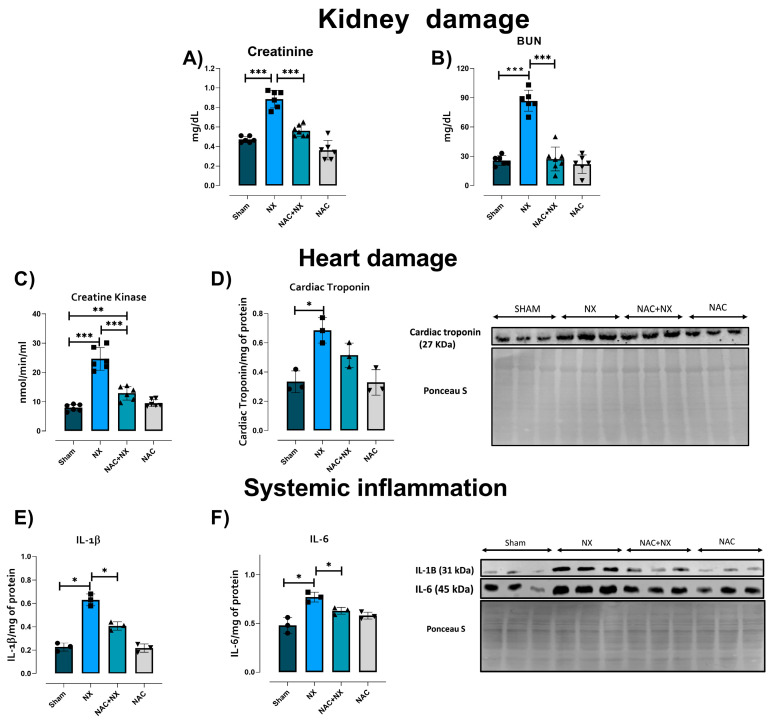
Cardio-renal syndrome development blood markers. Evaluation of the renal damage markers (**A**) creatinine and (**B**) blood urea nitrogen (BUN) at 10 days after surgery in plasma. The results are expressed in milligrams per deciliter (mg/dL). (**C**) Creatine Kinase and cardiac troponin (**D**) were evaluated as cardiac damage markers. Creatine kinase activity is expressed in nanomoles per minute per milliliter of plasma (nmol/min/mL). Plasma evaluation by Western blot of pro-inflammatory cytokines and their densitometry: (**E**) interleukin one beta (IL-1β) and (**F**) interleukin six (IL-6). Ponceau S staining of the corresponding membranes was used as a protein loading control. Data are the mean ± SEM, with n = 6–3. * *p* < 0.05, ** *p* < 0.01, and *** *p* < 0.001. NX = 5/6 nephrectomy. NAC = N-acetylcysteine.

**Figure 2:**
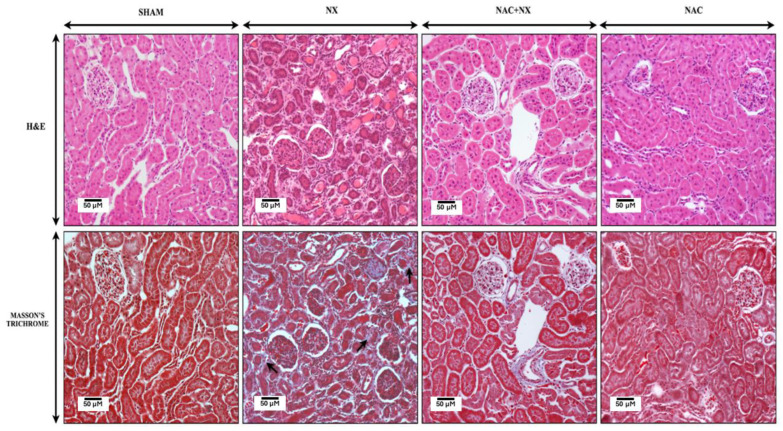
Representative micrographs of kidney histology. In comparison to the SHAM and NAC groups that show normal kidney histology, NX rats show small proximal convoluted tubules covered by a cuboidal or flat epithelium that corresponds to tubular atrophy; some of these tubules show hyaline cylinders in their lumen. There are focal interstitial chronic inflammatory infiltrates with slight fibrosis demonstrated in the Masson trichrome-stained section (arrow). These histological changes are clearly reduced in NAC+NX rats. NX = 5/6 nephrectomy. NAC = N-acetylcysteine.

To confirm kidney impairment, renal damage markers, namely creatinine (Figure 1A) and BUN (Figure 1B), were evaluated in plasma. As shown in Figure 1, both parameters were significantly increased in the NX group, as were kidney weight (KW) and the KW/body weight ratio (Table 1), confirming the presence of renal damage at 10 days. This is in agreement with previous works that showed a permanent increase in these markers from 24 h after surgery, which remained elevated throughout CKD progression and correlated with the progressive diminution in glomerular filtration rate (GFR) [21,22]. Furthermore, histology evaluation using H&E and Masson trichrome staining showed some areas with atrophy of proximal convoluted tubules in the NX group kidneys, manifested by flattened epithelial cells alternating with apoptotic and necrotic cells, and some tubules with hyaline casts in their lumen (Figure 2). Likewise, tubules and glomerulus in the NX group were surrounded by collagenous tissue (fibrosis) and chronic inflammatory infiltrates, differing from the Sham group (Figure 2). Arteries in these fibrotic areas showed thickened muscular wall layers.

While CRS type IV in the 5/6NX model has been reported at later stages after 2 months or more [32], studies in the early stages are scarce. To confirm the development of CRS type IV at this stage, creatine kinase activity and cardiac troponin levels in plasma were evaluated. Both markers showed a rise in the NX group (Figure 1C,D). CRS development has also been associated with higher plasma levels of cardio-renal connectors like pro-inflammatory cytokines and uremic toxins [25]. Interestingly, the NX group presented higher plasmatic IL-1β and IL-6 levels (Figure 1E,F), suggesting a systemic pro-inflammatory state in this group.

**Figure 3:**
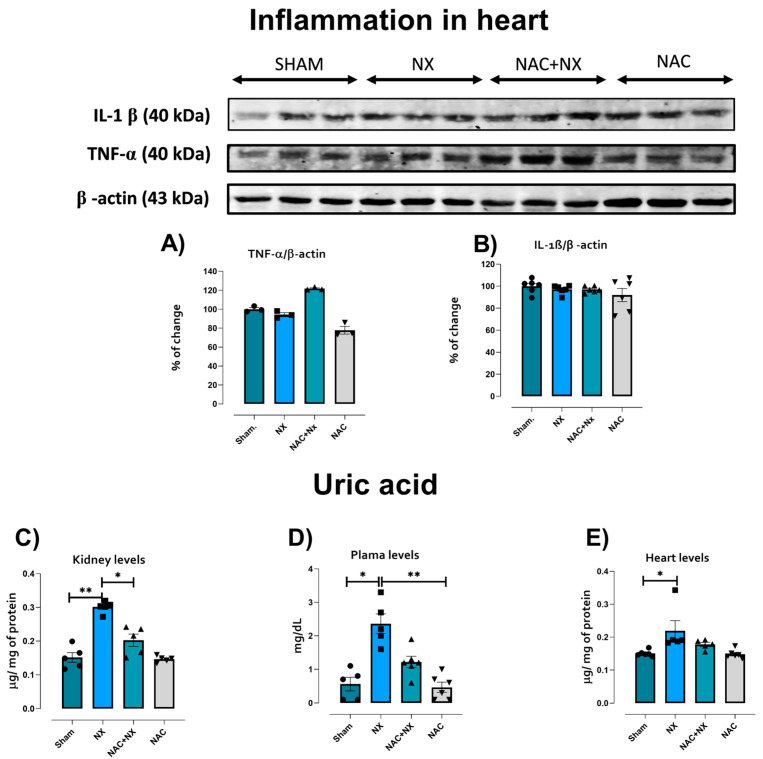
Inflammatory cytokines and uric acid levels. Evaluation of pro-inflammatory cytokines in heart tissue by Western blotting and their densitometry at 10 days after surgery: (**A**) interleukin one beta (IL-1β) and (**B**) tumor necrosis factor alpha (TNF-α). β-Actin of the corresponding membranes was used as a control. Evaluation of uric acid levels in (**C**) the kidneys, (**D**) plasma and (**E**) heart. Data are the mean ± SEM, with n = 5–6. * *p* < 0.05; ** *p* < 0.01. NX= 5/6 nephrectomy. NAC = N-acetylcysteine.

Evaluation of pro-inflammatory cytokines in the heart tissue homogenates did not show a significative increase at 10 days in the NX group (Figure 3A,B); however several reports showed that in the advanced stage of CRS, pro-inflammatory factors are overproduced in the heart [25]. Meanwhile, an early and significant increase in uric acid levels in kidneys and plasma was observed in the NX group (Figure 3C,D). This agrees with a previous report showing that uremic toxins are frequently found to be elevated in patients, promoting inflammation, oxidative stress and other pathological pathways that trigger CRS development [33].

**Figure 4:**
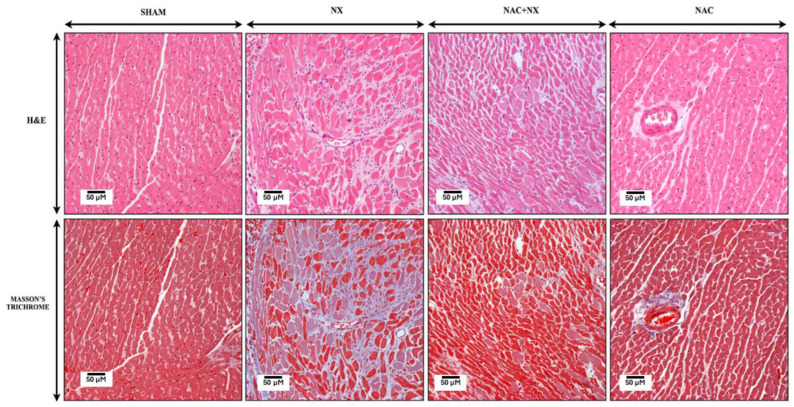
Representative micrographs of heart histology. In comparison to the SHAM and NAC groups that show normal heart histology, NX rats show undulating and fragmented myocytes surrounded by chronic inflammatory infiltrates and fibrosis, as seen in the Masson trichrome-stained section. These histological abnormalities are partially prevented in NAC+NX rats. NX = 5/6 nephrectomy. NAC = N-acetylcysteine.

**Figure 5:**
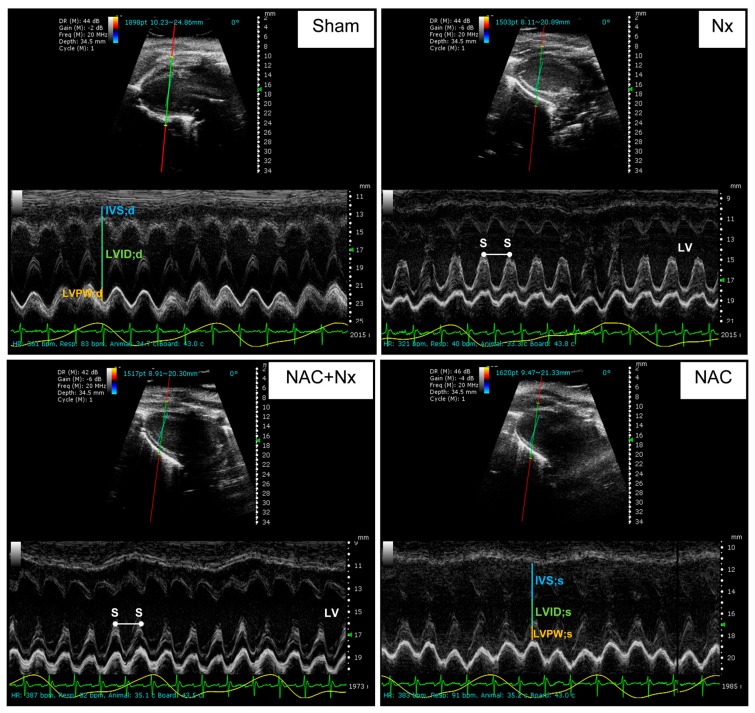
Echocardiography and functional cardiac parameters in rats. Representative paraesternal long-axis (SA) M-mode view with measurements of the left ventricular (LV) dimensions. With this parameter, it is possible to measure the LV internal diameter in diastole (LVID;d—yellow lines) and the LV internal diameter in systole (LVID;s—yellow lines). Left ventricular anterior wall thickness (LVAW) and posterior wall (LVPW) are shown in white and blue lines in diastole and systole. The heart rate is calculated using the distance between two consecutive systoles (S, white double-point arrow). NX= 5/6 nephrectomy; NAC = N-acetylcysteine.

Interestingly, uric acid also increased in the heart (Figure 3E), suggesting that uremic toxins increase before inflammation in this organ. Furthermore, histological analysis in NX group hearts by H&E and Masson trichrome staining showed focal groups of death or damaged undulated cardiomyocytes, with mild chronic inflammatory infiltrates around the damaged cells and blood vessels in comparison to the Sham group (Figure 4), confirming cardiomyocyte damage and the inflammatory response. Likewise, heart weight (HW) and the HW/tibia length ratio increased after 10 days in the NX group with respect to the Sham (Table 1), suggesting heart hypertrophy. Similarly, in NX group rats, systolic dysfunction was present, evidenced by fractional shortening (FS) and the ejection fraction (EF) being significantly lower compared to the Sham (Figure 5 and Table 2). Together, these results validate the early development of CRS-IV at 10 days.

Previously, our group demonstrated that NAC administration prevented CRS type III in a folic acid AKI model [25]. This is congruent with our current findings of notably lower renal damage markers in the NAC+NX group compared to NX (Figure 1A,B), reducing the inflammatory markers IL-1β and IL-6 in plasma (Figure 1E,F) and structural alterations such as inflammation and extracellular matrix deposition in the kidneys (Figure 2). Furthermore, NAC also prevented creatine kinase activity increases (Figure 1C), significantly reduced plasmatic and kidney uric acid increases (Figure 3C,D), and showed a tendency to reduce heart uric acid levels (Figure 3E), although this is not significant.

NAC pre-administration also prevented histological and inflammatory alterations, as observed by H&E and Masson trichrome staining, in the heart (Figure 4). In the case of cardiac function (Table 2), all other parameters were unchanged during diastole (Figure 5 and Table 2). The NAC+NX 5 group also demonstrated notable improvements in FS and the EF, reaching levels comparable to the Sham group (Table 2). Concerning stroke volume (SV) and end-systolic volume (ESV), NAC pretreatment enhanced both parameters to values like those observed in the Sham group (Figure 5 and Table 2). In contrast to SF and the EF, cardiac output (CO) and heart rate did not change between the experimental groups. Finally, NAC administration alone did not cause changes in any of the echocardiographic parameters (Table 2) or histology evaluation (Figure 4).

Collectively, these results suggest that NAC partially prevented CRS -IV development induced by NX in the early stages, reducing systemic inflammation and uric acid increases.

### 3.2. NAC Prevented Mitochondrial Bioenergetic Impairment in Kidney and Heart

**Figure 6:**
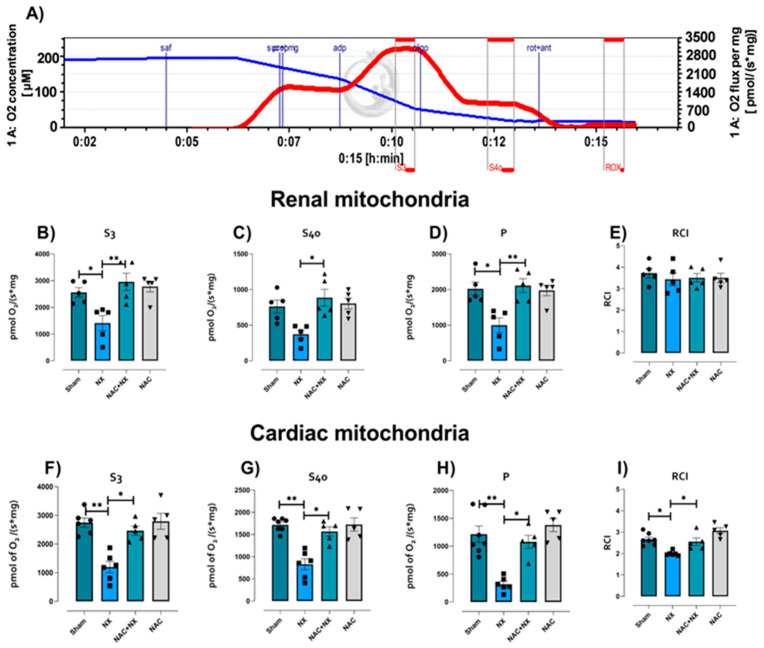
Respiratory parameters in isolated mitochondria from kidney and heart. (**A**) A representative graph of the method used to determine the CI and CI + CII-linked respiratory parameters in isolated mitochondria. The chamber O_2_ concentration is shown by the blue line, and respiratory rate is shown in red; the values are normalized per milligram of protein [pmol/(s*mg)]. Renal mitochondria respiratory parameters: (**B**) S3, (**C**) S4o, (**D**) P and (**E**) RCI. Respiratory parameters in isolated mitochondria in the heart: (**F**) S3, (**G**) S4o, (**H**) P and (**I**) RCI. Data are the mean ± SEM, with n = 5–6. * *p* < 0.05; ** *p* < 0.01. S3 = respiratory state 3, S4o = respiratory state 4 induced by oligomycin, P = respiration directly associated with OXPHOS, RCI = respiratory control index, CI = complex I, CII = complex II, PMG = pyruvate–malate-glutamate, S = succinate, Oligo = oligomycin, Rot + ant = rotenone plus antimycin A, and ROX = residual non-mitochondrial respiration. NX = 5/6 nephrectomy. NAC = N-acetylcysteine.

Mitochondrial homeostasis is essential for heart and renal metabolism [34], and thus impairment in this organelle triggers injury in both organs [34,35]. Mitochondrial bioenergetic dysfunction has emerged as an early factor that favors renal damage progression. To explore if the early kidney impairment observed at 10 days was related to a reduction in mitochondrial metabolism, the respiratory parameters assessed using high-resolution respirometry in renal-isolated mitochondria with CI + CII-linked substrates were evaluated (Figure 6A). The NX group showed lower values in respiratory states S3, S4o and P (Figure 6B–D) with respect to the Sham, implying a reduction in total OXPHOS capacity. Interestingly, no change was observed in RCI (Figure 6E), which suggests the absence of mitochondrial uncoupling 10 days after NX. These results are congruent with the reported reduction in mitochondrial respiration with β-oxidation-linked substrates during the first month after surgery [22], and suggest that mitochondrial OXPHOS is compromised in renal tissues regardless of substrate choice due to mitochondrial nephrons.

Recently, it has been demonstrated in CRS type III that heart mitochondria alteration is an early predictor of cardiac impairment [25]; therefore, we evaluated if heart mitochondria respiratory state was also altered at 10 days after nephrectomy. Interestingly, heart mitochondria in the NX group also showed lower S3 and *p* values, implying that cardiac OXPHOS capacity was also decreased (Figure 6F). Furthermore, S4o also decreased in comparison to the Sham group (Figure 6G), indicating that leak processes also increase in the heart, which may be associated with mitochondrial decoupling in this organ (Figure 6H).

**Figure 7:**
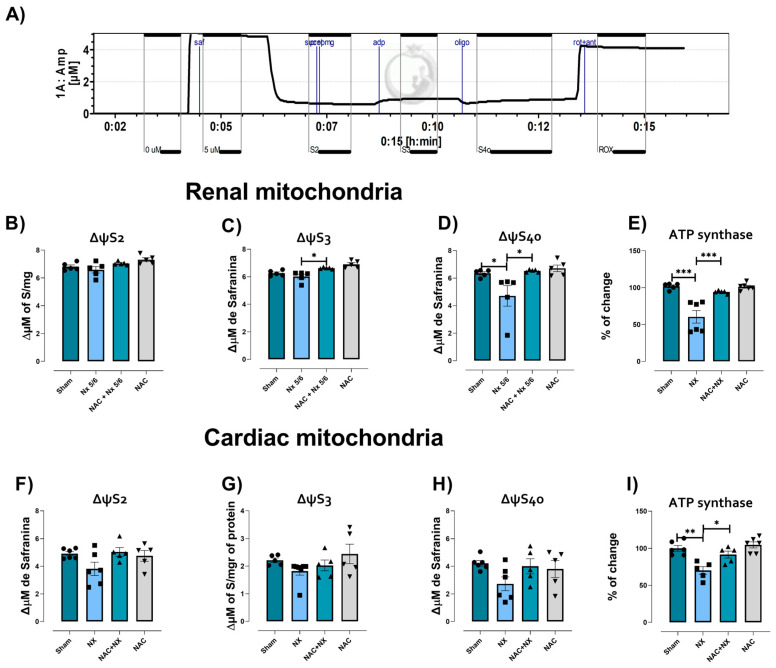
Evaluation of the changes in mitochondrial membrane potential (Δψm) and ATP synthase activity in isolated mitochondria. (**A**) Graphic representations of the changes in safranin O fluorescence used to evaluate the changes in Δψm. Δψm changes in (**B**) S2, (**C**) S3 and (**D**) S4o and (**E**) ATP synthase activity in renal mitochondria. Δψm changes in cardiac isolated mitochondria in the respiratory state, (**F**) S2, (**G**) S3 and (**H**) S4o, as well as (**I**) ATP synthase activity in cardiac mitochondria. Data are the mean ± SEM, with n = 5–6. * *p* < 0.05, ** *p* < 0.01, and *** *p* < 0.001. State 2 = S3, State 3 = S3, state 4 induced by oligomycin = S4o, PMG = pyruvate–malate-glutamate, S = succinate, Oligo = oligomycin, Rot + ant= rotenone plus antimycin A, and ROX = residual non-mitochondrial respiration. NX= 5/6 nephrectomy. NAC = N-acetylcysteine.

To relate the observed alteration in respiratory state with changes in ΔΨm, the latter was evaluated using safranine O as a probe in the different respiratory states (Figure 7A). NX significantly reduced ΔΨm in S3 and S4o in renal and cardiac mitochondria; however, this was only significant in S4o in renal mitochondria (Figure 7D). Moreover, ATP synthase activity was reduced in both renal and cardiac mitochondria (Figure 7E,I), suggesting that although mitochondria maintain ΔΨm after surgery (Figure 7B–H), ATP production by this organelle is strongly decreased in both cardiac and kidney tissue.

**Figure 8:**
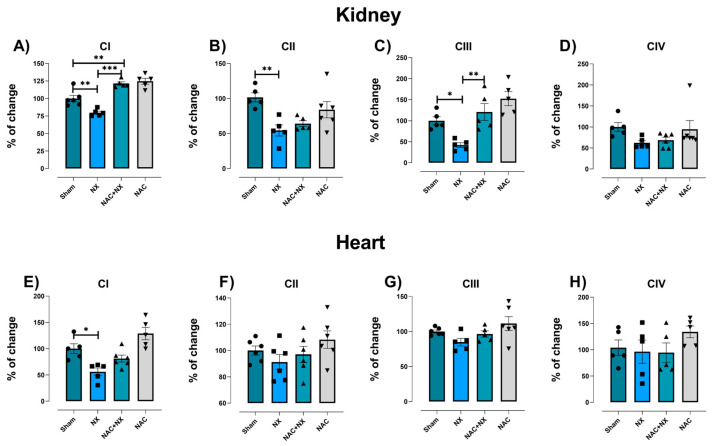
Mitochondrial electron transport complexes activity. The activity of renal electron transport system complexes: (**A**) CI, (**B**) CII, (**C**) CIII and (**D**) CIV. The activity of heart electron transport system complexes: (**E**) CI, (**F**) CII, (**G**) CIII and (**H**) CIV. Data are the mean ± SEM, with n = 5–6. * *p* < 0.05, ** *p* < 0.01, and *** *p* < 0.001. CI = complex I, CII = complex II, CIII = complex III, CIV = complex IV, NX = 5/6 nephrectomy, and NAC = N-acetylcysteine.

Interestingly, the separate evaluation of mitochondrial electron transport system complexes showed that after 10 days of NX, the activity of CI, CII and CIII was reduced, with no change in CIV activity in renal tissue (Figure 8A–D). This agrees with our previously reported findings that showed that CI and CIII sustain early damage in renal tissue following NX [22]. In this way, in the heart tissue, only CI activity was reduced in the NX group (Figure 8E), without significant changes in the rest of the mitochondrial complexes (Figure 8F–H). Together, our results suggest that in the kidney, OXPHOS reduction may be attributable to damage in several complexes; meanwhile, OXPHOS reduction in the heart may be primarily attributable to CI impairment.

NAC pretreatment prevented respiratory parameter alterations in isolated mitochondria from the kidneys (Figure 6B–D) and heart (Figure 6F–I), implying that NAC can restore OXPHOS capacity in both organs, as well as mitochondrial decoupling in the heart. This agrees with the ΔΨm restoration induced by NAC in renal mitochondria (Figure 7C,D), as well as the activity of ATP synthase in both tissues (Figure 7E,I). In renal tissue, the protective effects can be attributed to CI and CIII activity restoration induced by NAC (Figure 8A,C). Meanwhile, in the heart, CI activity in the NAC+NX group tended to increase, but this was not significant with respect to NX (Figure 8E). Interestingly, in CRS type III, NAC has also been reported to prevent CI activity loss in the heart due to high-dose folic acid administration [25]. Therefore, these results suggest that NAC administration also prevented renal and cardiac mitochondrial bioenergetic disturbance in nephrectomy-induced CRS-IV.

### 3.3. NAC Prevented Redox Imbalance in Renal and Heart

**Figure 9:**
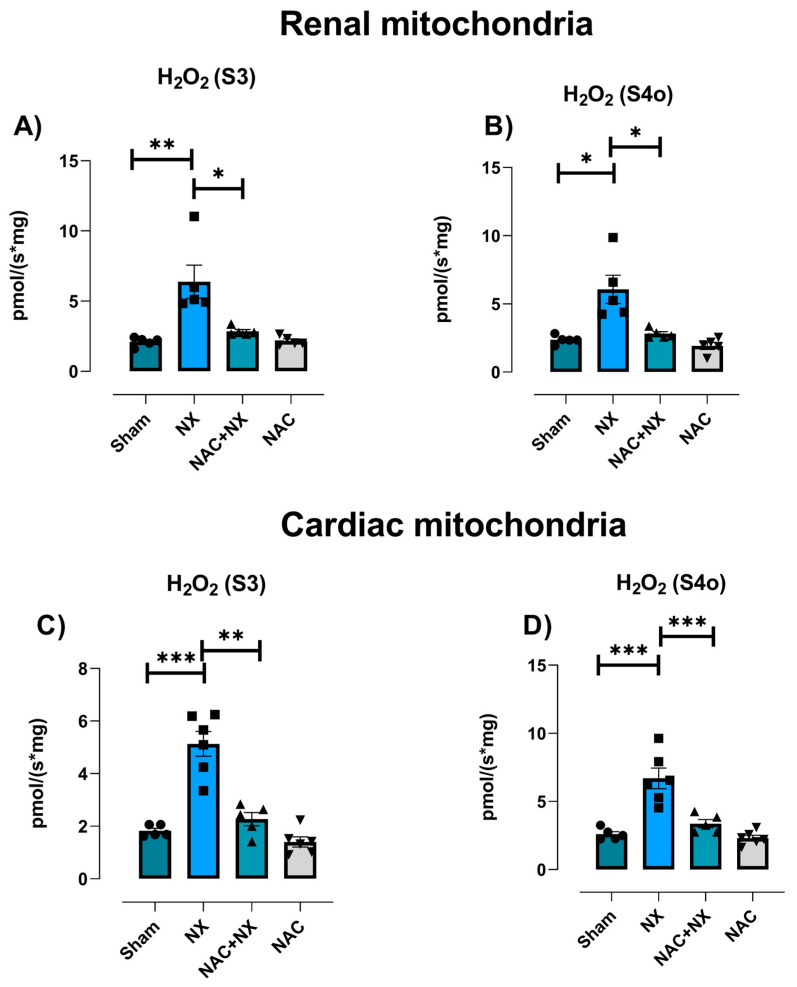
Mitochondrial hydrogen peroxide (H_2_O_2_) production. Renal mitochondria H_2_O_2_ production rates in CI + CII-linked respiration evaluated with an O2k-Fluorometer (OROBOROS, Innsbruck, Austria) using Amplex red as a probe in the respiratory state: (**A**) S3 and (**B**) S4o. Heart mitochondria H_2_O_2_ production rates in CI + CII-linked respiration in the respiratory state: (**C**) S3 and (**D**) S4o. Data are the mean ± SEM, with n = 5–6. * *p* < 0.05, ** *p* < 0.01, and *** *p* < 0.001. CI = complex I, CII = complex II, S3 = state 3, S4o= state 4 induced by olygomicin, NX = 5/6 nephrectomy, and NAC = N-acetylcysteine.

In CKD, mitochondrial respiration impairment has been linked with higher ROS production [28]. Likewise, reductions in renal and heart mitochondrial CI activity are associated with higher ROS production [25,26]. Thus, the mitochondrial H_2_O_2_ production rate in isolated mitochondria from both organs was evaluated. The NX group’s respiratory rates according to CI + CII-linked activity showed higher rates of H_2_O_2_ in S3 and S4o with respect to the Sham group (Figure 9A,B). Interestingly, heart mitochondria also showed higher rates of H_2_O_2_ production in both respiratory states (Figure 9C,D). These results suggest that NX induced an overproduction of ROS in high-energy-demand organs.

**Figure 10:**
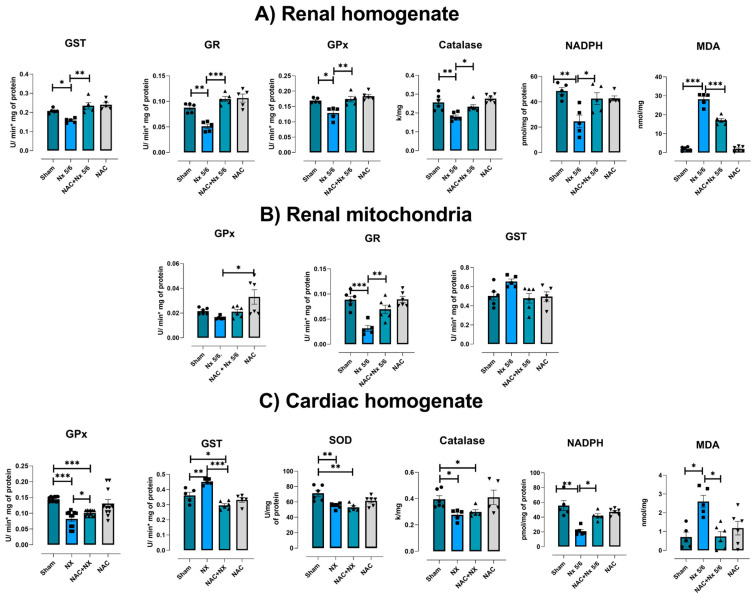
Oxidative stress in the kidneys and heart. Evaluation of the activity of antioxidant enzymes: GR = glutathione reductase, GPx = glutathione peroxidase, catalase, GST = glutathione S-transferase, and SOD = superoxide dismutase. The levels of NADPH (nicotinamide adenine dinucleotide phosphate) and the oxidative stress marker MDA (Malondialdehyde) were reduced as shown in (**A**) renal homogenates, (**B**) kidney isolated mitochondria and (**C**) heart homogenates. Data are the mean ± SEM, with n = 5–6. * *p* < 0.05, ** *p* < 0.01, and *** *p* < 0.001. NX = 5/6 nephrectomy. NAC = N-acetylcysteine.

To evaluate if the H_2_O_2_ increase was also accompanied by alterations in antioxidant enzymes, we evaluated the renal and heart activity of SOD, catalase, GPx, GR and GST, as well NADPH levels in total homogenates and isolated mitochondria from both organs. In renal homogenates, we observed a reduction in the activity of enzymes in the NX group, namely catalase, GPx, GR and GST enzymes, as well as in NAPDH levels (Figure 10A). Likewise, renal mitochondria from the NX group also showed a significant reduction in GR (Figure 10B), and a tendency for GPx activity to decrease and for GST to increase during this period. Meanwhile, the evaluation in heart homogenates showed lower SOD, catalase and GPx activities, lower NADPH levels, and higher MDA levels and GST enzyme activity in the NX group (Figure 10C). These results confirm that NX-induced CRS-IV triggers an early pro-oxidative state in the heart and kidneys.

NAC pretreatment significantly prevented the rise in mitochondrial H_2_O_2_ production in S3 and S4o in both kidney and heart mitochondria (Figure 9A–D). Furthermore, it prevented a decrease in the activities of catalase, GPx, GR and GST and in NADPH levels in renal homogenates (Figure 10A) and GR in kidney mitochondria (Figure 10B).

NAC antioxidant effects were also observed in heart homogenates, with alterations in GPx and GST activities and MDA and NADPH levels prevented, although there were no effects on SOD and catalase activities (Figure 10B). These results suggest that NAC offers redox protection present in the kidneys and has a partial protective effect in heart tissue.

### 3.4. NAC Prevents Mitochondrial Biogenesis Reduction in Kidney

CKD and CRS type III have been associated with changes in mitochondrial mass reduction resulting from biogenesis impairment [1,23,25,36]. Therefore, to determine if the observed impairment in renal and heart bioenergetics was related to changes in the SIRT/PGG-1α/NRF pathway, we evaluated the proteins related to biogenesis in kidney and heart tissues.

**Figure 11:**
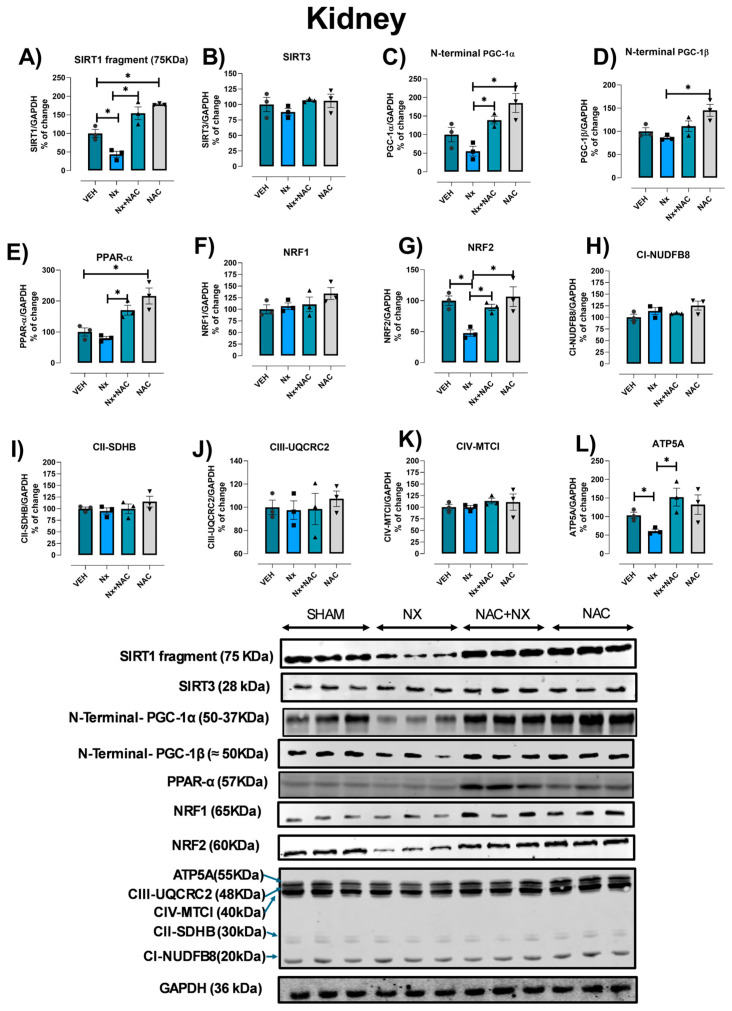
Mitochondrial biogenesis in renal tissue. Evaluation in kidney of mitochondrial biogenesis factors by Western blotting and their densitometry at 10 days after surgery: (**A**) SIRT1 fragment of 75 KDa, (**B**) SIRT3, (**C**) N-terminal-PGC-1α, (**D**) N-terminal-PGC-1β, (**E**) PPAR-α, (**F**) NRF1, (**G**) NRF2, (**H**) CI-NDUFB8, (**I**) CII-SDHB, (**J**) CIII-UQCRC2, (**K**) CIV-MTCO1 and (**L**) ATP5A. GAPDH of the corresponding membranes was used as a protein loading control. Data are the mean ± SEM, with n = 3. * *p* < 0.05. CI-NDUFB8 = reduced form of the nicotinamide adenine dinucleotide ubiquinone oxidoreductase subunit B8; CII-SDHB = iron-sulfur subunit B of succinate dehydrogenase complex; CIII-UQCRC2 = ubiquinol-cytochrome c reductase core protein 2; CIV-MTCO1 = cytochrome c oxidase subunit I, ATP5A = ATP synthase subunit α; GAPDH = glyceraldehyde-3-phosphate dehydrogenase; NRF1 = nuclear respiratory factor 1; NRF2 = nuclear respiratory factor 2; NX = 5/6 nephrectomy, NAC = N-acetyl-cysteine; PGC-1α = peroxisome proliferator-activated receptor gamma coactivator 1-alpha; PGC-1β = peroxisome proliferator-activated receptor gamma coactivator 1-beta; PPAR-α = peroxisome proliferator-activated receptor alpha; SIRT1 = sirtuin 1; SIRT3 = sirtuin 3.

Renal mitochondria biogenesis in the NX group after 10 days was strongly downregulated because of the decreased levels of the 75 KDa SIRT1 fragment (Figure 11A), N-terminal-PGC-1α (Figure 11D) and NRF2 (Figure 11G), resulting in lower levels of ATP5A subunits (Figure 11L). This partially explains the early decrease in OXPHOS capacity (Figure 6) and ATP synthase activity (Figure 7) of renal mitochondria from nephrectomy rats at 10 days after surgery. This result also agrees with our previous reports that showed a progressive decrease in mitochondrial renal content as time since surgery increased [23].

**Figure 12:**
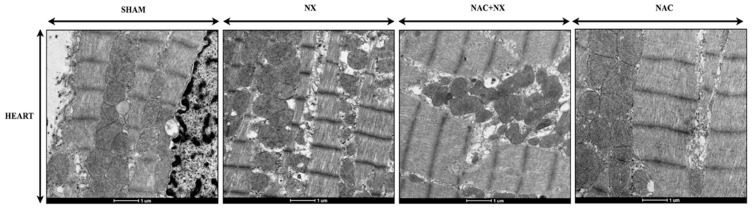
Representative electron micrographs of heart mitochondria. SHAM and NAC micrographs show the normal distribution and shape of mitochondria, while NX rats show swollen and fragmented mitochondria, and many are small or show an irregular shape with cristae effacement. These mitochondrial abnormalities are prevented in NX rats treated with NAC. NX = 5/6 nephrectomy. NAC = N-acetylcysteine.

Interestingly, 10 days after NX, heart tissues from the NX group did not show a reduction in mitochondrial biogenesis factors or OXPHOS subunit protein levels (Supplementary Figure S1), suggesting that in heart tissues, bioenergetic alterations (Figure 6, Figure 7 and Figure 8) are not yet associated with impairments in SIRT/PGC-1α/NRFs, as seen in other CRS models at more advanced stages [1,25]. Interestingly, the electron microscopy evaluation in the heart showed that in the NX group, there are swollen, fragmented and irregular-shaped mitochondria with cristae effacement, without evident changes in organelle number (Figure 12). These findings support the hypothesis that in the heart, early mitochondrial impairment is primarily linked with alterations in bioenergetics rather than changes in biogenesis.

On the other hand, NAC has been shown to induce mitochondrial biogenesis via SIRTs and PGC-1α pathway activation [1,26,37,38]. In this way, NAC pre-administration prevented the diminution in the 75 KDa SIRT1 fragment (Figure 11A), N-terminal-PGC-1α (Figure 11D) and NRF2 (Figure 11G), with a concomitant increase in ATP5A subunit levels (Figure 11L) in renal tissues induced by nephrectomy. Furthermore, NAC also significantly increased N-terminal-PGC-1β levels (Figure 11D) in renal homogenates compared to the Sham and NX groups, which suggests that this drug has positive effects on mitochondrial biogenesis in this model. Accordingly, NAC also increased PGC-1β and PPAR-α levels in the heart (Supplementary Figure S1) compared to the NX and Sham groups, with no perceptible improvement in mitochondrial mass observed in the NAC+NX group (Figure 12). This suggests that the overactivation of PGC-1β and PPAR-α is associated with the protective effect observed in heart mitochondria.

### 3.5. In Silico Activation of PPAR-α and NRF2 by NAC

We recently reported that NAC potentially activates mitochondrial biogenesis through its direct interaction with and activation of SIRT1 and SIRT3 [1]. However, the potential activation of NAC through direct interaction with other mitochondrial biogenesis factors remains unclear. Because the WB results showed that PPAR-α levels increased in the NAC-administrated group (Figure 11 and Figure 12), the possible activation of this transcription factor by NAC was explored using an in silico approach. It is important to investigate this approach with PPAR-α because, in both CKD and CRS, it plays a key role in the regulation of mitochondrial and lipid metabolism [39]; moreover, it is highly expressed in the heart and kidneys, because both tissues highly depend on mitochondrial fatty acid β-oxidation [40,41,42,43]. Furthermore, metabolic disorders like obesity and diabetes are linked with decreases in PPAR-α levels, prompting nephropathy and cardiomyopathy. Moreover, in experimental models and clinical studies, PPAR-α agonist (pemafibrate, bezafibrate, clofibrate) administration shows nephroprotective effects [40,44,45].

The in silico approach results showed the formation of a higher stable complex with the common ligand pemafibrate (ΔG = −10.6 Kcal/mol) in comparison with deprotoned NAC (ΔG = −4.3 Kcal/mol); nevertheless, both compounds share an interaction area between α-helices H3, H5, and H7, next to antiparallel β-sheet S2 (Supplementary Figure S2). This difference can be explained according to the intrinsic molecular area of each molecule, as pemafibrate (521.39 Å^2^) presented an area 10 times bigger than that of NAC^−^(159.14 Å^2^). However, one important factor that supports the efficient interaction of PPAR-α−NAC^−^ is the minimum difference in the polar surface area (PSA) [46] between pemafibrate and NAC^−^, 62.839 Å and 56.005 Å, respectively, due to the presence of polar atoms in both molecules. Therefore, it is predicted that both compounds are similarly accessible like agonists of PPAR-α (Supplementary Figure S2C,D).

Likewise, NRF2, also known as GA-binding protein (GABP), is a transcription factor that binds to DNA and regulates the gene expression of mitochondrial proteins, genes that regulate the cell cycle, protein synthesis and cellular metabolism [47,48,49]. NRF2 is a heterodimer of two GABPα and two GABPβ polipeptides; the first binds to DNA via interaction with the Erythroblast Transformation Specific domain, while sa secondary structure is formed by antiparallel β-sheet (S1–S4) surrounded by four α-helices (H1–H4). On the other hand, GABPβ has protein–protein interactions with GABPα through a 4–5 ankyrin-repeat motif, which is essential for heterodimerization with GABPα. NRF2 activation and binding to DNA in turn occur after its binding to the PGC-1α peptide [48,50]; for this reason, this peptide has been used as a control in the molecular docking analysis. Furthermore, according to the literature, resveratrol could be an NRF2 agonist, supporting its use as another ligand positive control [47,50].

The in silico approach results revealed (Supplementary Figure S3) that NAC^−^ and resveratrol interact with NRF2 in the same region (interface GABPα and DNA). In the NAC^−^−NRF2 complex (ΔG = −3.54 Kcal/mol), the main force of union was Van der Waals interactions with Trp36, Try380, Try381, Met386, Gly384 and Trp323; these interactions allowed for effective binding with DNA and GABPα (Supplementary Figure S3A–D).

In the case of resveratrol−NRF2 (ΔG = −6.38 Kcal/mol), hydrogen bonds formed between Asp385 and the OH group in the phenol ring were detected; also, it was possible to identify two hydrophilic interactions with DNA (A31 y C32). PGC-1α (ΔG = −6.60 Kcal/mol) showed an interaction area near to that of H1 and H4 in the upper part of the 4–5 ankyrin-repeat motif (Thr65, Met77, Thr39, Leu44, Arg15, Tyr15, Leu406, Cys401, Asp402, Leu403). This type of interaction could explain the activation of NRF2. Furthermore, PGC-1α did not disrupt the GABPβ-GABPα interaction. The difference in the union energy could be explained by differences in the PSA and intrinsic molecular area of each molecule.

Together, these results suggest that NAC may activate mitochondrial biogenesis by binding to PPAR-α and NRF2, similarly to its suggested direct interaction with SIRT1 and SIRT3 [1].

## 4. Discussion

The global prevalence of CKD has reached 9.1%, making it one of the diseases with the fastest annual growth [7,51]. It is also a major factor associated with CRS type IV prevalence. Moreover, 70% of end-stage renal disease patients develop cardiac dysfunction [3,4,5]. Although, the CKD pathophysiology is multifaceted, and the damage propagation pathways affecting the heart are still poorly understood [1,5,52]. Several cardio-renal connectors like hemodynamic alterations, inflammatory response and damage-associated molecular pattern (DAMPs) production, fibrosis, renin–angiotensin–aldosterone system (RAAS) overactivation, extracellular vesicle production, redox and metabolic imbalance, lipotoxicity, accumulation of uremic toxins and others are currently under study [1].

In this respect, mitochondrial imbalance has recently emerged as a hub in the regulation of many of these cardio-renal connectors, being central in the pathophysiology of CRS development [1,34,53]. In CRS-IV, experimental studies have shown that in advanced stages of the disease, cardiac mitochondria suffer a decrease in their ATP production capacity, increasing oxidative stress and inflammation in the heart [27,32,54]. However, mitochondrial alterations occur in the early stages of CRS-IV. Its temporal development remains understudied, as do its contribution to the pathophysiology mechanisms.

This work aimed to characterize the first cardiac mitochondrial alterations after kidney damage. As demonstrated in Figure 6, both renal and cardiac mitochondria have a significant reduction in P and S3 states at 10 days after nephrectomy, implying a decrease in mitochondrial OXPHOS capacity. This is associated with lower CI activity in both tissues (Figure 8), favoring a decrease in ATP synthase activity (Figure 7). This is in agreement with our previous studies in which we showed that mitochondrial ATP production in the kidneys is lower at 24 h after surgery, remaining the same throughout CKD progression, and decreasing mitochondrial β-oxidation [21,22]. Since β-oxidation in the renal proximal tubule supports gluconeogenesis, which is essential for ATP production in glycolytic segments [43], renal mitochondrial impairment compromises the bioenergetic balance in all nephron segments [22]. Likewise, lower β-oxidation is associated with the impairment of the electron transfer system (ETS) [22], especially in CI activity, as observed in Figure 8.

Renal mitochondrial bioenergetic imbalance is linked to increased production of ROS by this organelle (Figure 9), as well as renal imbalance in the redox state (Figure 10A,B), favoring cell death and inflammatory infiltration in renal tissues (Figure 2). An additional consequence is the increase in the concentration of uremic toxins such as uric acid, whose levels rise early in the kidneys and plasma (Figure 3), acting as a contributing factor that further enhances the inflammation, oxidative stress and mitochondrial impairment in the kidneys [55,56].

Moreover, the release of damaged mitochondrial components like mtDAMPs triggers inflammatory responses via activation of the nucleotide-binding domain-like receptor family pyrin domain-containing protein 3 (NLRP3) inflammasome and the nuclear factor-kappa B (NF-κB) pathways [34,35], which augments the renal release of inflammatory cytokines. In this respect, we observed an early increase in IL-1β and IL-6 plasma levels (Figure 3). Furthermore, it was previously reported that renal CI and CIII inhibition induced in CKD is associated with NLRP3 and NF-κB pathway activation [22,57]. These findings suggest that the observed renal ETS impairment (Figure 8) can promote the release of cardio-renal connectors (Figure 1, Figure 2 and Figure 3).

It has been widely described in CKD that the progressive decline in renal function is associated with the rise in uremic toxins [55,56]. Thus, in models of hypouricemic nephropathy, uric acid enhances renal NLRP3 activation, affecting mitochondrial balance in the heart [58]. Likewise, the increase in uric acid and related pathways like the purine degradation pathway has been described to induce metabolic reprogramming, decreasing mitochondrial coupling and ATP production [56]. The increase in intracellular uric acid concentration induces translocation of the ROS-producing enzyme NADPH oxidase (NOX) to the mitochondria, favoring oxidative stress and calcium deregulation, inducers of endoplasmic reticulum stress [59,60]. This would be particularly important in our model, where there is an early rise in the heart uric acid concentration (Figure 3E), which could trigger heart mitochondria to reduce the coupling degree and OXPHOS capacity (Figure 6 and Figure 7). Such effects trigger mitochondrial ROS production enhancement, leading to cardiac oxidative stress, as observed (Figure 9 and Figure 10).

In agreement, it has been reported that other uremic toxins like IS trigger mitochondrial impairment and heart complications in AKI and CKD [14,61,62]. In particular, uremic toxin administration enhanced cardiac ROS levels and reduced mitochondrial biogenesis [17,18], which was also observed in our model in the early stages (Figure 10 and Figure 12). Uremic toxins are also related to heart hypertrophy (Figure 4, Table 1 and Table 2) and inflammation (Figure 4). In fact, the increase in plasma uremic toxins was associated with left ventricular hypertrophy and cardiac NLRP3 and NF-κB pathway activation in CKD patients [63,64,65]. Interestingly, our results showed that CKD induces a small heart hypertrophy at 10 days after surgery, with systolic dysfunction and a lower EF (Figure 5 and Table 2).

We recently reported that NX induces a decrease in mitochondrial ATP production, redox imbalance in the heart triggering CRS-IV progression after 2 months [27]. To our knowledge, this represents an initial investigation aiming to characterize the cardiac mitochondrial impairment (Figure 6, Figure 7 and Figure 8) occurring 10 days post NX surgery. This impairment is shown in bioenergetic parameters such as respiratory state and ΔΨm (Figure 6, Figure 7 and Figure 8). Therefore, our findings demonstrate that mitochondrial bioenergetic damage is an early mechanism that promotes the development of CRS type IV.

**Figure 13:**
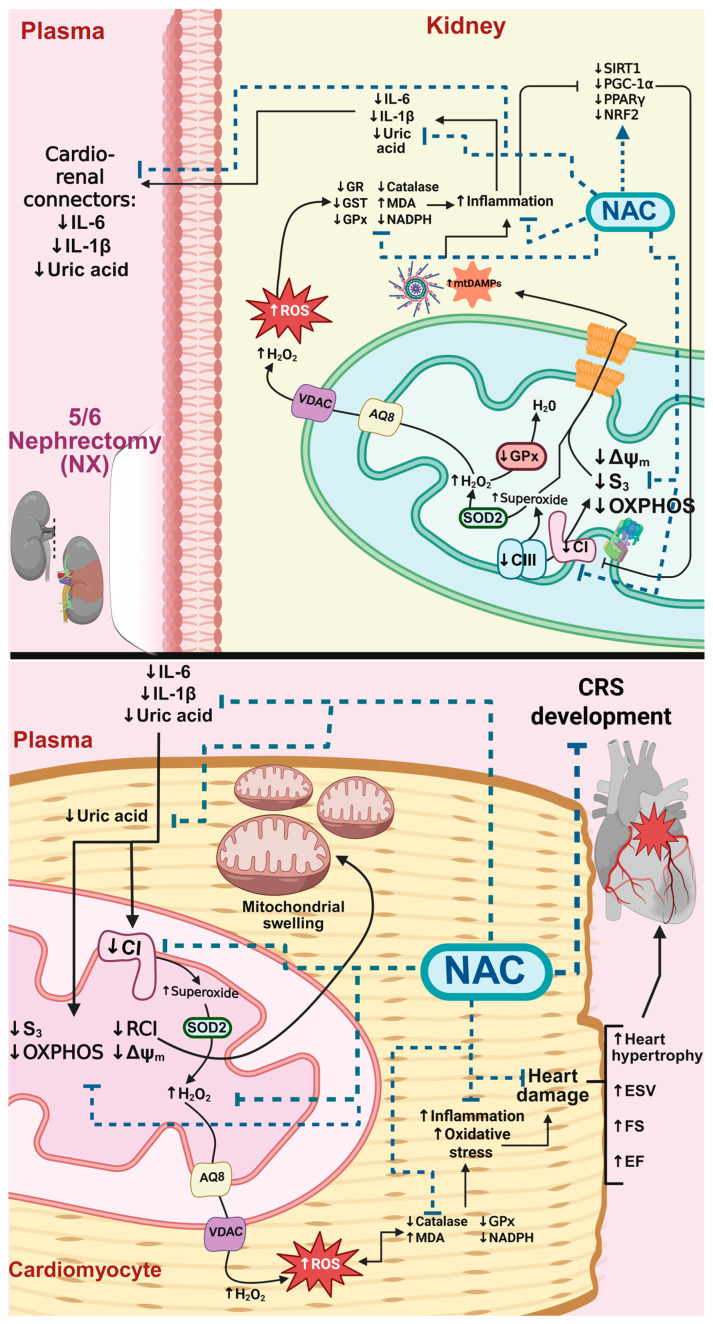
Integrative Scheme. 5/6Nx after 10 days in renal tissue induces mitochondrial bioenergetic impairment, characterized by the initial reduction in CI, CIII and ATP synthase activities, thus triggering S3 and OXPHOS reduction, as a result of lower Δψm. Mitochondrial complex impairment augments the rate of H_2_O_2_ production by this organelle, which together with the decrease in antioxidant enzyme capacity and redox balance promotes the mitochondrial release of mtDAMPS and higher ROS levels in cytosol. Thus, bioenergetic and redox impairment in renal tissue promotes a reduction in the regulators of the mitochondrial biogenesis AMPK-SIRT1/3-PGC-1α pathway, promoting an increase in the inflammatory pathways and the release from the kidneys to the blood stream of pro-inflammatory cytokines and uric acid, which act as cardio-renal connectors. In the early stages after renal injury induction, the cardio-renal connectors promote cardiac mitochondrial reduction in CI activity, which reduces OXPHOS capacity and promotes mitochondrial decoupling and swelling, also enhancing H_2_O_2_ production by this organelle. Like the kidneys, mitochondrial impairment promotes cytosol oxidative increases and inflammation increases in cardiac tissue. Although significant, the AMPK-SIRT1/3-PGC-1α pathway was not observed in the early stages after renal injury. The bioenergetics and redox alterations by themselves can induce functional impairment of the heart, leading to CRS development. In contrast, NAC showed protective effects in mitochondrial homeostasis in both tissues. In the kidneys, NAC protection can be linked to the protection of bioenergetics, the redox state and the activation of the AMPK-SIRT1/3-PGC-1α pathway, possibly via direct activation of some of its regulatory proteins. In contrast, in the heart, at this stage, NAC only presents protective effects on bioenergetic and redox parameters, without significantly promoting cardiac mitochondrial biogenesis. However, NAC effects on mitochondria in both tissues are capable of significantly preventing early CRS progression in this model. AQ8 = aquaporin eight, CI = complex I, CIII = complex III, CRS = cardio renal syndrome, EF = ejection fraction, ESV= end-systolic volume, FS= fractional shortening, GPx = glutathione peroxidase, GR= glutathione reductase, GST = glutathione S-transferase, H_2_O_2_ = hydrogen peroxide, IL-1β = interleukin one beta, IL-6 = interleukin six, MDA= Malondialdehyde, mtDAMPs = mitochondrial damage-associated patrons, NRF1 = nuclear respiratory factor 1, NRF2= nuclear respiratory factor 2, NX= 5/6 nephrectomy, NAC = N-acetyl-cysteine, NADPH= nicotinamide adenine dinucleotide phosphate reduced, PGC-1α = peroxisome proliferator-activated receptor gamma coactivator 1-alpha, PGC-1β = peroxisome proliferator-activated receptor gamma coactivator 1-beta, PPAR-α = peroxisome proliferator-activated receptor alpha, OXPHOS = oxidative phosphorylation, RCI = respiratory control index, ROS= reactive oxygen species, SIRT1 = sirtuin 1, SIRT3 = sirtuin 3, SOD2 = superoxide dismutase 2, State 3 = S3, S4o= state 4 induced by oligomycin, VDAC = voltage depending anion channel, and Δψm = mitochondrial membrane potential.

On the other hand, the inflammatory processes involving NFKB and NLRP3 pathways have also been associated with the inhibition of renal mitochondrial biogenesis by SIRT/PGC-1α/NRFs in diabetic nephropathy and CKD transition [66,67,68]. Our results showed that in the kidneys at 10 days after nephrectomy, the 75 KDa SIRT1 fragment, PPAR-α and NRF2 were significantly diminished (Figure 11), which may have contributed to the observed bioenergetic imbalance in this organ. In contrast, the evaluation at this time in heart tissue (Supplementary Figure S1) did not show a significant reduction in these factors, which correlated with the electron microscopy analysis where no changes in mitochondrial mass were perceptible (Figure 13). These results suggest that mitochondrial biogenesis impairment did not contribute to mitochondrial bioenergetic alterations in the heart, at least in this early stage. In contrast, we recently reported a decrease in SIRT3 levels in the heart at 2 months after NX, which indeed correlate with cardiac damage [27] and the reported decrease in SIRT1, SIRT3, PGC1-α and NRF-2 in the heart at advanced stages of CRS-IV [69]. Therefore, this suggests that the sustained presence of a pro-inflammatory environment and bioenergetic impairment ultimately results in decreased biogenesis. Consequently, reduced mitochondrial biogenesis in the heart acts as a subsequent factor that exacerbates mitochondrial damage, further diminishing OXPHOS capacity.

On the other hand, NAC administration has been employed in both experimental models and clinic studies to prevent oxidative stress, inflammation, fibrosis and bioenergetic alterations in AKI and CKD [70,71,72,73,74]. Interestingly, NAC administration has been more effective in pre- or parallel treatment to the induction of renal damage, with acute or short-term administration being sufficient to maintain or restore GSH plasma and tissue concentrations without pathological side effects [75]. In this regard, our previous studies demonstrated that NAC pre-administration prevented CKD transition due to high levels of folic acid [28]. Likewise, the present results show that NAC early administration reduced the increase in clinical markers of renal damage (Figure 1) and histological alterations in the kidneys (Figure 2). In the kidneys, NAC protective effects have been linked to the morphological preservation of the glomerulus and proximal tubule [26,28,76,77], as observed by histology (Figure 2). Likewise, NAC also reduced ROS, inflammation levels and cell death in the kidneys [26,28,72,78,79,80]. This is particularly relevant, because previous studies showed that NAC also decreases the release of cytokines and uremic toxins to the circulation [24,27,38,81,82,83].

Thus, the observed NAC protection of the redox state in kidney tissue (Figure 9 and Figure 10), is responsible for the reduction in uric acid and interleukins in plasma with respect to NX (Figure 1 and Figure 3). In fact, NAC can reduce the renal activation of inflammatory pathways mediated by the NLRP3 inflammasome and NF-κB, which are overactivated by mitochondrial impairment in renal tissues [79,84,85,86]. Consequently, as observed in the pre-administrated group, NAC redox state restoration is associated with the preservation of mitochondrial membrane potential and the activity of CI and CIII (Figure 7 and Figure 8). In this regard, we previously showed in folic acid-induced AKI to CKD transition that CI and CIII preservation by NAC is linked to the mitochondrial GSH/GSSG ratio and S-glutathionylation restoration in kidney mitochondria [26,28]. In fact, several authors showed that redox-sensitive post-translational modifications like S-glutathionylation represent a link between ROS balance and bioenergetic regulation in this organelle [25,26,28,87].

Although GSH/GSSG ratio restoration is identified as a key factor involved in the protective effects of NAC on renal tissue mitochondria, recent evidence suggests that NAC can also enhance other mitochondrial pathways, primarily the induction of biogenesis mediated by SIRT1/3-PGC-1α. NAC also activates AMPK, an upstream PGC-1α activator, which leads to SIRT3 phosphorylation in CKD. Likewise, NAC promotes SIRT3-dependent deacetylation, promoting the activation of several biogenesis factors [88,89]. Similarly, several ETS complexes and Krebs cycle enzymes are activated by NAC-Sirt3 pathways [25,90]. In agreement with our results, NAC pre-administration prevented a decrease in SIRT1, PGC-1α and NRF2 levels in the kidneys (Figure 11). Furthermore, NAC also increased PPAR-α and N-terminal-PGC-1β renal levels (Figure 11), implying overactivation in mitochondrial biogenesis. This increase has been reported in early stages in the AKI to CKD transition after NAC administration [25,26], suggesting that acute or short-term administration of NAC is particularly effective in promoting renal mitochondrial biogenesis. NAC-induced AMPK-SIRT1/3-PGC-1α overactivation is particularly important, because it has been reported to diminish mitochondrial ROS production and NLRP3 and TGF-β inflammasome activation in AKI and CKD [25,68,91,92,93]. This leads to the observed reduction in cytokine and uric acid release to the blood stream (Figure 1 and Figure 3), preventing cardiac damage development.

In this regard, it has been suggested that that NAC can activate proteins involved in AMPK-SIRT1/3-PGC-1α by directly interacting with them [1,94]. It was reported that NAC has binding region sites for SIRT3 like honokiol (an SIRT3 activator). Furthermore, NAC is also associated with resveratrol (an SIRT1 activator) in Sirt1 structures. In fact, the SIRT1-NAC complex has a ΔG = −3.1 kcal/mol, showing a favorable interaction. Meanwhile, in the case of SIRT3, NAC interacted near the hydrophilic cavity occupied by Honokiol with a ΔG = −4.6 kcal/mol, suggesting the possibility that NAC acts as suirtin activator [1,94]. Our results also showed that NAC is similarly accessible in the nucleus membrane of PPAR-α compared to pemafibrate, an agonist of PPAR-α with a ΔG = −4.3 Kcal/mol of the NAC-PPAR-α complex (Supplementary Figure S2), suggesting the possibility that NAC may act as an agonist. Likewise, the in silico approach results also showed that NAC can interact with NRF2 in the same region as resveratrol (Supplementary Figure S3), a molecule that enhances its interaction with DNA [47,50]. This interaction has ΔG = −3.54 Kcal/mol, suggesting that NAC may also promote the NRF2 transcriptional antibody. Although these predicted interactions have not been proven in chemical or crystallography experiments, many experimental studies showed that NAC administration restores and enhances mitochondrial biogenesis and antioxidant enzyme expression in renal damage models [25,90]. Thus, the potential direct activation of proteins in the AMPK-SIRT1/3-PGC-1α pathway may explain these experimental observations, as well as the reduction in inflammatory pathways.

However, several studies have shown that NAC has protective effects in cardiac surgery and heart tissues in CRS-III and IV [1,25]. Our understanding of how mitochondrial preservation contributes to these protective effects remains limited, especially in the early stages after kidney damage induction. We previously demonstrated that NAC prevented cardiac ETS activity reduction and oxidative stress induced by folic acid CRS-III [25]. In agreement with our results, NAC pretreatment prevented the reduction in respiratory parameters in the heart (Figure 6F–I), restoring OXPHOS capacity and mitochondrial coupling. As in folic acid-induced CRS-III, the sustained OXPHOS capacity in the NAC pre-administered group is related to cardiac ΔΨm maintenance (Figure 7), due to CI activity preservation and lower H_2_O_2_ mitochondrial production (Figure 8 and Figure 9). Although, unlike the kidney, the effects of NAC pre-administration do not appear to be linked to AMPK-SIRT1/3-PGC-1α pathway activation, except for the observed increase in PGC-1β and PPAR-α in the heart (Figure 12), the effects of preventing cardiac oxidative stress are evident in the pretreated group (Figure 10).

As illustrated in the integrative schema (Figure 13), these findings suggest that maintaining cardiac mitochondrial bioenergetics is crucial for preventing the alterations in cardiac function and hypertrophy observed in the 10-day NX group. Consequently, both recent studies and our research propose that NAC-mediated mitochondrial protection may represent a promising approach for preventing AKI to CKD progression and cardio-renal syndrome development.

## 5. Study Limitations

The objectives of this study were focused on finding the earliest time at which heart mitochondrial respiration impairment appears and to evaluate NAC’s protective effect against mitochondrial damage in CRS development. One significant limitation of our study is the fact that both mitochondrial damage and NAC’s protective effects throughout the development of CRS were not evaluated. In fact, it must be considered that a subsequent sustained post-administration regimen of NAC would be necessary to have a sustained effect on CRS development [27]. Another important limitation of this study lies in the short-term experimental endpoint of mitochondrial impairment evaluation. Although several studies showed that single-nephron glomerular filtration rate, renal hemodynamics, inflammation and metabolic alterations are already present at this stage [95,96,97,98,99,100], it should be highlighted that other relevant pathological processes that also contribute to the development of CRS, such as uremia, lipotoxicity, liver damage, extensive inflammatory process and hypertension, are not yet present at this stage [1,9,22,101,102,103,104,105]. Several of these factors, like lipotoxicity, permanent inflammation and uremic toxins, have been reported to induce significant changes on bioenergetics and redox balance [1,17,18,19,20], so their effects on the temporal evolution of the observed mitochondrial alterations should not be overlooked. Thus, it is still necessary to carry out a more extended temporal evaluation of mitochondrial dynamics, bioenergetic and biogenesis in the heart, as well as its correlation with inflammatory processes. In the case of mitochondrial dynamics, it is worth highlighting that previous studies have shown a sustained decrease in the kidneys [23]. However, there are no temporal studies in the heart. In this regard, our results show changes in a fragment form of SIRT1. Although the relevance of SIRT1 fragment form in in this model has not been elucidated, it was reported that it is involved in pro-inflammatory cytokine-induced apoptosis regulation [106]. As our study represents only the evolution stage, it is still necessary to conduct a temporal evaluation of mitochondrial biogenesis factors like SIRT1 and its correlation with inflammatory and cell death processes in CRS evolution.

## 6. Conclusions

This study demonstrated that mitochondrial bioenergetic and redox alterations are early developments after kidney damage induction, affecting not only the kidneys but also the heart. In the heart, OXPHOS reduction and redox imbalance may be triggered by increased levels of cardio-renal connectors in plasma, such as uric acid and pro-inflammatory cytokines. These factors lead to a diminution in CI activity and membrane depolarization, favoring an increase in heart mitochondria ROS production that leads to heart inflammation, hypertrophy and functional impairment. Thus, our results point to mitochondrial damage as one of the early central pathways in the development of CRS-IV. Furthermore, we showed that the early preservation of renal and heart mitochondria by NAC administration could be a useful strategy to prevent the onset of CRS-IV. Interestingly, NAC preserves not only the redox and bioenergetic mitochondrial balance in both renal and kidney tissue but also induces overactivation of the SIRT1/3-PGC-1α pathway and its downstream effectors, mainly in the kidneys. Although the effects of NAC were linked to increased levels of proteins such as SIRT1, PPAR and NRF2, in vitro results suggest that NAC may also directly interact with and activate several of these factors, suggesting opportunities for future studies exploring the nature and mechanisms of these interactions.

## Figures and Tables

**Table 1:** Morphometric parameters.

Parameter	Sham	NX	NAC+NX	NAC
BW (g)	229 ± 19	242 ± 9.9	235 ± 18	249 ± 25
HW (g)	0.93 ± 0.13	1.2 ± 0.13 *	1.2 ± 0.11 *	1.2 ± 0.14 *
LW (g)	1.6 ± 0.39	2.1 ± 0.17	2.1 ± 0.32	2.2 ± 0.55
KW (g)	0.89 ± 0.09	1.2 ± 0.18 *	1.2 ± 0.12 *	1 ± 0.11
HW/BW (g/Kg)	4 ± 0.27	4.9 ± 0.37	5.1 ± 0.56 *	4.7 ± 0.67
LW/BW (g/Kg)	7.1 ± 1.6	8.8 ± 0.71	9.1 ± 1.6	8.8 ± 2.6
KW/BW (g/Kg)	3.9 ± 0.48	5 ± 0.67 *	5.1 ± 0.65 *	4.2 ± 0.17 **
HW/TL (g/cm)	0.19 ± 0.03	0.25 ± 0.02 *	0.25 ± 0.03 *	0.27 ± 0.04 *
LW/TL (g/cm)	0.34 ± 0.08	0.45 ± 0.04	0.45 ± 0.07	0.49 ± 0.14 *
KW/TL (g/cm)	0.18 ± 0.02	0.25 ± 0.03 *	0.25 ± 0.03 *	0.24 ± 0.01 *

BW = body weight; HW = heart weight; LW = lung weight; KW = kidney weight; TL = tibial length. Data are mean ± SD, n = 5–8. * *p* ≤ 0.05 vs. Sham; ** *p* ≤ 0.05 vs. NX. NX = 5/6 nephrectomy, NAC = N-acetylcysteine.

**Table 2:** Cardiac parameters obtained from the echocardiography analysis.

Parameter	Sham	NX	NAC+NX	NAC
HR (bpm)	355.9 ± 19.3	365.2 ± 23.5	391 ± 24.6	365.9 ± 28.9
IVS;d (mm)	0.9 ± 0.1	1.1 ± 0.2	1.1 ± 0.3	1.1 ± 0.3
LVID;d (mm)	6.9 ± 0.8	6.7 ± 0.7	6 ± 0.6	6.8 ± 0.7
LVPW;d (mm)	1.5 ± 0.3	1.2 ± 0.2	1.3 ± 0.3	1.2 ± 0.1
FS (%)	57.8 ± 14.2	38.5 ± 7.9 *	52 ± 9.9 ***	52.1 ± 5.5
EDV (µL)	253.5 ± 65.9	236.2 ± 52.4	181.7 ± 39.9	245.6 ± 52.9
ESV (µL)	42.4 ± 37.1	78.2 ± 28.3	35.3 ± 17.5 ***	48.3 ± 18.8
EF (%)	84.3 ± 10.1	66.5 ± 9.5 *^,^**	80.1 ± 9.6 ***	81.1 ± 4.6
SV (µL)	211.1 ± 42.9	158 ± 44.1	146.4 ± 36.2 *	197.3 ± 36
CO (mL/min)	76.7 ± 17.7	57.2 ± 15.4	57.5 ± 15.1	72 ± 14

Abbreviations: d: diastole; HR: heart rate; bpm: beats per minute; IVS: interventricular septum; LVID: LV internal diameter in diastole; LVPW: LV posterior wall; FS: fractional shortening; EDV: end-diastolic volume; ESV: end-systolic volume; EF: ejection fraction; SV: stroke volume; CO: cardiac output; mm: millimeters; μL: microliters. Data are the mean ± SD, with n = 5–8. * *p* ≤ 0.05 vs. Sham; ** *p* ≤ 0.05 vs. NAC; *** *p* ≤ 0.05 vs. NX. NX = 5/6 nephrectomy. NAC = N-acetylcysteine.

## Data Availability

Data are contained within the article.

## Supplementary Materials

The following supporting information can be downloaded at: https://www.mdpi.com/article/10.3390/antiox14101241/s1, Figure S1: Evaluation in heart of mitochondrial biogenesis factors by Western blot and its densitometry at 10 days after surgery; Figure S2: Ribbons representation of the complex PPAR-α (PDB code: 6KB4) with pemafibrate and NAC; Figure S3: Ribbons representation of the complex NRF2 (PDB code: 1AWC) with PGC-1α; Figure S4: Evaluation in plasma and heart of proinflammatory cytokines; Table S1: List of probes, antibodies, reagents and consumables used, as well as their catalog numbers and manufacturing companies.

## References

[1] Lumpuy-Castillo J., Amador-Martínez I., Díaz-Rojas M., Lorenzo O., Pedraza-Chaverri J., Sánchez-Lozada L.G., Aparicio-Trejo O.E. (2024). Role of Mitochondria in Reno-Cardiac Diseases: A Study of Bioenergetics, Biogenesis, and GSH Signaling in Disease Transition. Redox Biol..

[2] Szlagor M., Dybiec J., Młynarska E., Rysz J., Franczyk B. (2023). Chronic Kidney Disease as a Comorbidity in Heart Failure. Int. J. Mol. Sci..

[3] Xue Y., Xu B., Su C., Han Q., Wang T., Tang W. (2019). Cardiorenal Syndrome in Incident Peritoneal Dialysis Patients: What Is Its Effect on Patients’ Outcomes?. PLoS ONE.

[4] Prothasis M., Varma A., Gaidhane S., Kumar S., Khatib N., Zahiruddin Q., Gaidhane A. (2020). Prevalence, Types, Risk Factors, and Outcomes of Cardiorenal Syndrome in a Rural Population of Central India: A Cross-Sectional Study. J. Fam. Med. Prim. Care.

[5] Suresh H., Arun B.S., Moger V., Swamy M. (2017). Cardiorenal Syndrome Type 4: A Study of Cardiovascular Diseases in Chronic Kidney Disease. Indian Heart J..

[6] Yu A.S., Pak K.J., Zhou H., Shaw S.F., Shi J., Broder B.I., Sim J.J. (2023). All-Cause and Cardiovascular-Related Mortality in CKD Patients With and Without Heart Failure: A Population-Based Cohort Study in Kaiser Permanente Southern California. Kidney Med..

[7] (2020). GBD Chronic Kidney Disease Collaboration Global, Regional, and National Burden of Chronic Kidney Disease, 1990-2017: A Systematic Analysis for the Global Burden of Disease Study 2017. Lancet.

[8] Bhargava P., Schnellmann R.G. (2017). Mitochondrial Energetics in the Kidney. Nat. Rev. Nephrol..

[9] Hamzaoui M., Djerada Z., Brunel V., Mulder P., Richard V., Bellien J., Guerrot D. (2020). 5/6 Nephrectomy Induces Different Renal, Cardiac and Vascular Consequences in 129/Sv and C57BL/6JRj Mice. Sci. Rep..

[10] Bigelman E., Cohen L., Aharon-Hananel G., Levy R., Rozenbaum Z., Saada A., Keren G., Entin-Meer M. (2018). Pathological Presentation of Cardiac Mitochondria in a Rat Model for Chronic Kidney Disease. PLoS ONE.

[11] Ryan J., Treberg J.R. (2016). Protein S-Glutathionlyation Links Energy Metabolism to Redox Signaling in Mitochondria. Redox Biol..

[12] Singh C.K., Chhabra G., Ndiaye M.A., Garcia-Peterson L.M., MacK N.J., Ahmad N. (2018). The Role of Sirtuins in Antioxidant and Redox Signaling. Antioxid. Redox Signal..

[13] Ceballos-Picot I., Witko-Sarsat V., Merad-Boudia M., Nguyen A.T., Thévenin M., Jaudon M.C., Zingraff J., Verger C., Jungers P., Descamps-Latscha B. (1996). Glutathione Antioxidant System as a Marker of Oxidative Stress in Chronic Renal Failure. Free Radic. Biol. Med..

[14] Popkov V.A., Silachev D.N., Zalevsky A.O., Zorov D.B., Plotnikov E.Y. (2019). Mitochondria as a Source and a Target for Uremic Toxins. Int. J. Mol. Sci..

[15] Shen W.C., Chou Y.H., Shi L.S., Chen Z.W., Tu H.J., Lin X.Y., Wang G.J. (2021). Ast-120 Improves Cardiac Dysfunction in Acute Kidney Injury Mice via Suppression of Apoptosis and Proinflammatory Nf-Κb/Icam-1 Signaling. J. Inflamm. Res..

[16] Tan X., Cao X.S., Zhang P., Xiang F.F., Teng J., Zou J.Z., Ding X.Q. (2018). Endoplasmic Reticulum Stress Associated Apoptosis as a Novel Mechanism in Indoxyl Sulfate-Induced Cardiomyocyte Toxicity. Mol. Med. Rep..

[17] Enoki Y., Watanabe H., Arake R., Fujimura R., Ishiodori K., Imafuku T., Nishida K., Sugimoto R., Nagao S., Miyamura S. (2017). Potential Therapeutic Interventions for Chronic Kidney Disease-Associated Sarcopenia via Indoxyl Sulfate-Induced Mitochondrial Dysfunction. J. Cachexia Sarcopenia Muscle.

[18] Sun C.Y., Cheng M.L., Pan H.C., Lee J.H., Lee C.C. (2017). Protein-Bound Uremic Toxins Impaired Mitochondrial Dynamics and Functions. Oncotarget.

[19] Koizumi M., Tatebe J., Watanabe I., Yamazaki U., Ikeda T., Morita T. (2014). Aryl Hydrocarbon Receptor Mediates Indoxyl Sulfate-Induced Cellular Senescence in Human Umbilical Vein Endothelial Cells. J. Atheroscler. Thromb..

[20] Nakagawa K., Itoya M., Takemoto N., Matsuura Y., Tawa M., Matsumura Y., Ohkita M. (2021). Indoxyl Sulfate Induces ROS Production via the Aryl Hydrocarbon Receptor-NADPH Oxidase Pathway and Inactivates NO in Vascular Tissues. Life Sci..

[21] Aparicio-Trejo O.E., Tapia E., Molina-Jijón E., Medina-Campos O.N., Macías-Ruvalcaba N.A., León-Contreras J.C., Hernández-Pando R., García-Arroyo F.E., Cristóbal M., Sánchez-Lozada L.G. (2017). Curcumin Prevents Mitochondrial Dynamics Disturbances in Early 5/6 Nephrectomy: Relation to Oxidative Stress and Mitochondrial Bioenergetics. BioFactors.

[22] Aparicio-Trejo O.E., Rojas-Morales P., Avila-Rojas S.H., León-Contreras J.C., Hernández-Pando R., Jiménez-Uribe A.P., Prieto-Carrasco R., Sánchez-Lozada L.G., Pedraza-Chaverri J., Tapia E. (2020). Temporal Alterations in Mitochondrial β-Oxidation and Oxidative Stress Aggravate Chronic Kidney Disease Development in 5/6 Nephrectomy Induced Renal Damage. Int. J. Mol. Sci..

[23] Prieto-Carrasco R., García-Arroyo F.E., Aparicio-Trejo O.E., Rojas-Morales P., León-Contreras J.C., Hernández-Pando R., Sánchez-Lozada L.G., Tapia E., Pedraza-Chaverri J. (2021). Progressive Reduction in Mitochondrial Mass Is Triggered by Alterations in Mitochondrial Biogenesis and Dynamics in Chronic Kidney Disease Induced by 5/6 Nephrectomy. Biology.

[24] Khan S.A., Campbell A.M., Lu Y., An L., Alpert J.S., Chen Q.M. (2021). N-Acetylcysteine for Cardiac Protection During Coronary Artery Reperfusion: A Systematic Review and Meta-Analysis of Randomized Controlled Trials. Front. Cardiovasc. Med..

[25] Cuevas-López B., Romero-Ramirez E.I., García-Arroyo F.E., Tapia E., León-Contreras J.C., Silva-Palacios A., Roldán F.-J., Campos O.N.M., Hernandez-Esquivel L., Marín-Hernández A. (2023). NAC Pre-Administration Prevents Cardiac Mitochondrial Bioenergetics, Dynamics, Biogenesis, and Redox Alteration in Folic Acid-AKI-Induced Cardio-Renal Syndrome Type 3. Antioxidants.

[26] Aparicio-Trejo O.E., Reyes-Fermín L.M., Briones-Herrera A., Tapia E., León-Contreras J.C., Hernández-Pando R., Sánchez-Lozada L.G., Pedraza-Chaverri J. (2019). Protective Effects of N-Acetyl-Cysteine in Mitochondria Bioenergetics, Oxidative Stress, Dynamics and S-Glutathionylation Alterations in Acute Kidney Damage Induced by Folic Acid. Free Radic. Biol. Med..

[27] Amador-Martínez I., Aparicio-Trejo O.E., Aranda-Rivera A.K., Bernabe-Yepes B., Medina-Campos O.N., Tapia E., Cortés-González C.C., Silva-Palacios A., Roldán F.J., León-Contreras J.C. (2025). Effect of N-Acetylcysteine in Mitochondrial Function, Redox Signaling, and Sirtuin 3 Levels in the Heart During Cardiorenal Syndrome Type 4 Development. Antioxidants.

[28] Aparicio-Trejo O.E., Avila-Rojas S.H., Tapia E., Rojas-Morales P., León-Contreras J.C., Martínez-Klimova E., Hernández-Pando R., Sánchez-Lozada L.G., Pedraza-Chaverri J. (2020). Chronic Impairment of Mitochondrial Bioenergetics and β-Oxidation Promotes Experimental AKI-to-CKD Transition Induced by Folic Acid. Free Radic. Biol. Med..

[29] Ojuka E., Andrew B., Bezuidenhout N., George S., Maarman G., Madlala H.P., Mendham A., Osiki P.O. (2016). Measurement of β-Oxidation Capacity of Biological Samples by Respirometry: A Review of Principles and Substrates. Am. J. Physiol. Metab..

[30] Morris G.M., Huey R., Lindstrom W., Sanner M.F., Belew R.K., Goodsell D.S., Olson A.J. (2009). AutoDock4 and AutoDockTools4: Automated Docking with Selective Receptor Flexibility. J. Comput. Chem..

[31] Eberhardt J., Santos-Martins D., Tillack A.F., Forli S. (2021). AutoDock Vina 1.2.0: New Docking Methods, Expanded Force Field, and Python Bindings. J. Chem. Inf. Model..

[32] Hernández-Reséndiz S., Correa F., García-Niño W.R., Buelna-Chontal M., Roldán F.J., Ramírez-Camacho I., Delgado-Toral C., Carbó R., Pedraza-Chaverrí J., Tapia E. (2015). Cardioprotection by Curcumin Post-Treatment in Rats with Established Chronic Kidney Disease. Cardiovasc. Drugs Ther..

[33] Chaudhary K., Malhotra K., Sowers J., Aroor A. (2013). Uric Acid—Key Ingredient in the Recipe for Cardiorenal Metabolic Syndrome. Cardiorenal Med..

[34] Shi S., Zhang B., Li Y., Xu X., Lv J., Jia Q., Chai R., Xue W., Li Y., Wang Y. (2022). Mitochondrial Dysfunction: An Emerging Link in the Pathophysiology of Cardiorenal Syndrome. Front. Cardiovasc. Med..

[35] Keshavarz-Bahaghighat H., Darwesh A.M., Sosnowski D.K., Seubert J.M. (2020). Mitochondrial Dysfunction and Inflammaging in Heart Failure: Novel Roles of CYP-Derived Epoxylipids. Cells.

[36] Stallons L.J., Whitaker R.M., Schnellmann R.G. (2014). Suppressed Mitochondrial Biogenesis in Folic Acid-Induced Acute Kidney Injury and Early Fibrosis. Toxicol. Lett..

[37] Dong W., Zhang K., Gong Z., Luo T., Li J., Wang X., Zou H., Song R., Zhu J., Ma Y. (2023). N-Acetylcysteine Delayed Cadmium-Induced Chronic Kidney Injury by Activating the Sirtuin 1–P53 Signaling Pathway. Chem. Biol. Interact..

[38] Li C., Xie N., Li Y., Liu C., Hou F.F., Wang J. (2019). N-Acetylcysteine Ameliorates Cisplatin-Induced Renal Senescence and Renal Interstitial Fibrosis through Sirtuin1 Activation and P53 Deacetylation. Free Radic. Biol. Med..

[39] Montaigne D., Butruille L., Staels B. (2021). PPAR Control of Metabolism and Cardiovascular Functions. Nat. Rev. Cardiol..

[40] Hu P., Li K., Peng X., Kan Y., Li H., Zhu Y., Wang Z., Li Z., Liu H.Y., Cai D. (2023). Nuclear Receptor PPARα as a Therapeutic Target in Diseases Associated with Lipid Metabolism Disorders. Nutrients.

[41] Rofaeil R.R., Abdellah A.M., Zenhom N.M. (2019). Nephroprotective Effect of PPAR Agonists on Thioacetamide-Induced Nephrotoxicity in Rats. J. Pharmacol. Clin. Res..

[42] Bernardes A., Souza P.C.T., Muniz J.R.C., Ricci C.G., Ayers S.D., Parekh N.M., Godoy A.S., Trivella D.B.B., Reinach P., Webb P. (2013). Molecular Mechanism of Peroxisome Proliferator-Activated Receptor α Activation by WY14643: A New Mode of Ligand Recognition and Receptor Stabilization. J. Mol. Biol..

[43] Kamata S., Oyama T., Saito K., Honda A., Yamamoto Y., Suda K., Ishikawa R., Itoh T., Watanabe Y., Shibata T. (2020). PPARα Ligand-Binding Domain Structures with Endogenous Fatty Acids and Fibrates. iScience.

[44] Rosas-Martínez L., Rodríguez-Muñoz R., Namorado-Tonix del Carmen M., Missirlis F., del Valle-Mondragón L., Sánchez-Mendoza A., Reyes-Sánchez J.L., Cervantes-Pérez L.G. (2024). Peroxisome Proliferator-Activated Receptor Alpha Stimulation Preserves Renal Tight Junction Components in a Rat Model of Early-Stage Diabetic Nephropathy. Int. J. Mol. Sci..

[45] Gao J., Gu Z. (2022). The Role of Peroxisome Proliferator-Activated Receptors in Kidney Diseases. Front. Pharmacol..

[46] Barret R. (2018). Importance and Evaluation of the Polar Surface Area (PSA and TPSA). Medicinal Chemistry.

[47] Canning P., Sorrell F.J., Bullock A.N. (2015). Structural Basis of Keap1 Interactions with Nrf2. Free Radic. Biol. Med..

[48] George M., Reddy A.P., Reddy P.H., Kshirsagar S. (2024). Unraveling the NRF2 Confusion: Distinguishing Nuclear Respiratory Factor 2 from Nuclear Erythroid Factor 2. Ageing Res. Rev..

[49] Kim M.-J., Jeon J.-H., Fernandez-Alfonso S., Gil-Ortega M., Kim M.-J., Jeon J.-H. (2022). Recent Advances in Understanding Nrf2 Agonism and Its Potential Clinical Application to Metabolic and Inflammatory Diseases. Int. J. Mol. Sci..

[50] Baldelli S., Aquilano K., Ciriolo M.R. (2013). Punctum on Two Different Transcription Factors Regulated by PGC-1α: Nuclear Factor Erythroid-Derived 2-like 2 and Nuclear Respiratory Factor 2. Biochim. Biophys. Acta Gen. Subj..

[51] Carrero J.-J., Hecking M., Ulasi I., Sola L., Thomas B. (2017). Chronic Kidney Disease, Gender, and Access to Care: A Global Perspective. Semin. Nephrol..

[52] Rangaswami J., Bhalla V., Blair J.E.A., Chang T.I., Costa S., Lentine K.L., Lerma E.V., Mezue K., Molitch M., Mullens W. (2019). Cardiorenal Syndrome: Classification, Pathophysiology, Diagnosis, and Treatment Strategies: A Scientific Statement From the American Heart Association. Circulation.

[53] Aroor A.R., Mandavia C., Ren J., Sowers J.R., Pulakat L. (2012). Mitochondria and Oxidative Stress in the Cardiorenal Metabolic Syndrome. Cardiorenal Med..

[54] Correa F., Buelna-Chontal M., Hernández-Reséndiz S., García-Niño W.R., Roldán F.J., Soto V., Silva-Palacios A., Amador A., Pedraza-Chaverrí J., Tapia E. (2013). Curcumin Maintains Cardiac and Mitochondrial Function in Chronic Kidney Disease. Free Radic. Biol. Med..

[55] Moradi H., Sica D.A., Kalantar-Zadeh K. (2013). Cardiovascular Burden Associated with Uremic Toxins in Patients with Chronic Kidney Disease. Am. J. Nephrol..

[56] García-Arroyo F.E., Monroy-Sánchez F., Muñoz-Jiménez I., Gonzaga G., Andrés-Hernando A., Zazueta C., Juárez-Rojas J.G., Lanaspa M.A., Johnson R.J., Sánchez-Lozada L.G. (2019). Allopurinol Prevents the Lipogenic Response Induced by an Acute Oral Fructose Challenge in Short-Term Fructose Fed Rats. Biomolecules.

[57] Swanson K.V., Deng M., Ting J.P.Y. (2019). The NLRP3 Inflammasome: Molecular Activation and Regulation to Therapeutics. Nat. Rev. Immunol..

[58] Zhang C., Song Y., Chen L., Chen P., Yuan M., Meng Y., Wang Q., Zheng G., Qiu Z. (2022). Urolithin A Attenuates Hyperuricemic Nephropathy in Fructose-Fed Mice by Impairing STING-NLRP3 Axis-Mediated Inflammatory Response via Restoration of Parkin-Dependent Mitophagy. Front. Pharmacol..

[59] Lanaspa M.A., Sanchez-Lozada L.G., Choi Y.J., Cicerchi C., Kanbay M., Roncal-Jimenez C.A., Ishimoto T., Li N., Marek G., Duranay M. (2012). Uric Acid Induces Hepatic Steatosis by Generation of Mitochondrial Oxidative Stress: Potential Role in Fructose-Dependent and -Independent Fatty Liver. J. Biol. Chem..

[60] Choi Y.J., Shin H.S., Choi H.S., Park J.W., Jo I., Oh E.S., Lee K.Y., Lee B.H., Johnson R.J., Kang D.H. (2014). Uric Acid Induces Fat Accumulation via Generation of Endoplasmic Reticulum Stress and SREBP-1c Activation in Hepatocytes. Lab. Investig..

[61] Zwaenepoel B., De Backer T., Glorieux G., Verbeke F. (2024). Predictive Value of Protein-Bound Uremic Toxins for Heart Failure in Patients with Chronic Kidney Disease. ESC Heart Fail..

[62] Caillard P., Bennis Y., Six I., Bodeau S., Kamel S., Choukroun G., Maizel J., Titeca-Beauport D. (2022). The Role of Gut-Derived, Protein-Bound Uremic Toxins in the Cardiovascular Complications of Acute Kidney Injury. Toxins.

[63] Yang K., Wang C., Nie L., Zhao X., Gu J., Guan X., Wang S., Xiao T., Xu X., He T. (2015). Klotho Protects against Indoxyl Sulphate-Induced Myocardial Hypertrophy. J. Am. Soc. Nephrol..

[64] Capomolla S., Opasich C., Riccardi G., Febo O., Riccardi R., Cobelli F., Tavazzi L. (1998). Beta Blockade Therapy in Chronic Heart Failure: Diastolic Function and Mitral Regurgitation Improvement by Carvedilol. J. Am. Coll. Cardiol..

[65] Dou L., Sallée M., Cerini C., Poitevin S., Gondouin B., Jourde-Chiche N., Fallague K., Brunet P., Calaf R., Dussol B. (2015). The Cardiovascular Effect of the Uremic Solute Indole-3 Acetic Acid. J. Am. Soc. Nephrol..

[66] Li F., Chen Y., Li Y., Huang M., Zhao W. (2020). Geniposide Alleviates Diabetic Nephropathy of Mice through AMPK/SIRT1/NF-ΚB Pathway. Eur. J. Pharmacol..

[67] Yuan Y., Huang S., Wang W., Wang Y., Zhang P., Zhu C., Ding G., Liu B., Yang T., Zhang A. (2012). Activation of Peroxisome Proliferator-Activated Receptor-γ Coactivator 1α Ameliorates Mitochondrial Dysfunction and Protects Podocytes from Aldosterone-Induced Injury. Kidney Int..

[68] Zhao W.Y., Zhang L., Sui M.X., Zhu Y.H., Zeng L. (2016). Protective Effects of Sirtuin 3 in a Murine Model of Sepsis-Induced Acute Kidney Injury. Sci. Rep..

[69] Huang Y., Wang S., Zhou J., Liu Y., Du C., Yang K., Bi X., Liu M., Han W., Wang K. (2020). IRF1-Mediated Downregulation of PGC1α Contributes to Cardiorenal Syndrome Type 4. Nat. Commun..

[70] Ergin B., Guerci P., Zafrani L., Nocken F., Kandil A., Gurel-Gurevin E., Demirci-Tansel C., Ince C. (2016). Effects of N-Acetylcysteine (NAC) Supplementation in Resuscitation Fluids on Renal Microcirculatory Oxygenation, Inflammation, and Function in a Rat Model of Endotoxemia. Intensive Care Med. Exp..

[71] Shimizu M.H.M., Gois P.H.F., Volpini R.A., Canale D., Luchi W.M., Froeder L., Heilberg I.P., Seguro A.C. (2017). N-Acetylcysteine Protects against Star Fruit-Induced Acute Kidney Injury. Ren. Fail..

[72] Shen Y., Miao N.J., Xu J.L., Gan X.X., Xu D., Zhou L., Xue H., Zhang W., Lu L.M. (2016). N-Acetylcysteine Alleviates Angiotensin II-Mediated Renal Fibrosis in Mouse Obstructed Kidneys. Acta Pharmacol. Sin..

[73] Ware K., Qamri Z., Ozcan A., Satoskar A.A., Nadasdy G., Rovin B.H., Hebert L.A., Nadasdy T., Brodsky S.V. (2013). N-Acetylcysteine Ameliorates Acute Kidney Injury but Not Glomerular Hemorrhage in an Animal Model of Warfarin-Related Nephropathy. Am. J. Physiol. Ren. Physiol..

[74] Kizilgun M., Poyrazoglu Y., Oztas Y., Yaman H., Cakir E., Cayci T., Akgul O.E., Kurt Y.G., Yaren H., Kunak Z.I. (2011). Beneficial Effects of N-Acetylcysteine and Ebselen on Renal Ischemia/Reperfusion Injury. Ren. Fail..

[75] Ye M., Lin W., Zheng J., Lin S. (2021). N-Acetylcysteine for Chronic Kidney Disease: A Systematic Review and Meta-Analysis. Am. J. Transl. Res..

[76] Machado J.T., Iborra R.T., Fusco F.B., Castilho G., Pinto R.S., Machado-Lima A., Nakandakare E.R., Seguro A.C., Shimizu M.H., Catanozi S. (2014). N-Acetylcysteine Prevents Endoplasmic Reticulum Stress Elicited in Macrophages by Serum Albumin Drawn from Chronic Kidney Disease Rats and Selectively Affects Lipid Transporters, ABCA-1 and ABCG-1. Atherosclerosis.

[77] Pereira L.V.B., Shimizu M.H.M., Rodrigues L.P.M.R., Leite C.C., Andrade L., Seguro A.C. (2012). N-Acetylcysteine Protects Rats with Chronic Renal Failure from Gadolinium-Chelate Nephrotoxicity. PLoS ONE.

[78] Mohamed N.A., Hassan M.H., Saleem T.H., Mohamed S.A., El-Zeftawy M., Ahmed E.A., Mostafa N.A.M., Hetta H.F., Al Shaimaa H., Abdallah A.A.M. (2022). KIM-1 and GADDI-153 Gene Expression in Paracetamol-Induced Acute Kidney Injury: Effects of N-Acetylcysteine, N-Acetylmethionine, and N-Acetylglucosamine. Turkish J. Biochem..

[79] Song L., Yao S., Zheng D., Xuan Y., Li W. (2022). Astaxanthin Attenuates Contrast-Induced Acute Kidney Injury in Rats via ROS/NLRP3 Inflammasome. Int. Urol. Nephrol..

[80] Gu Y., Huang F., Wang Y., Chen C., Wu S., Zhou S., Hei Z., Yuan D. (2018). Connexin32 Plays a Crucial Role in ROS-Mediated Endoplasmic Reticulum Stress Apoptosis Signaling Pathway in Ischemia Reperfusion-Induced Acute Kidney Injury. J. Transl. Med..

[81] Wang S., Liu G., Jia T., Wang C., Lu X., Tian L., Yang Q., Zhu C. (2022). Protection Against Post-Resuscitation Acute Kidney Injury by N-Acetylcysteine via Activation of the Nrf2/HO-1 Pathway. Front. Med..

[82] Huang S., You J., Wang K., Li Y., Zhang Y., Wei H., Liang X., Liu Y. (2019). N -Acetylcysteine Attenuates Cisplatin-Induced Acute Kidney Injury by Inhibiting the C5a Receptor. Biomed Res. Int..

[83] Liu C., Shen Y., Huang L., Wang J. (2022). TLR2/Caspase-5/Panx1 Pathway Mediates Necrosis-Induced NLRP3 Inflammasome Activation in Macrophages during Acute Kidney Injury. Cell Death Discov..

[84] Amador-Martínez I., Aparicio-Trejo O.E., Bernabe-Yepes B., Aranda-Rivera A.K., Cruz-Gregorio A., Sánchez-Lozada L.G., Pedraza-Chaverri J., Tapia E. (2023). Mitochondrial Impairment: A Link for Inflammatory Responses Activation in the Cardiorenal Syndrome Type 4. Int. J. Mol. Sci..

[85] Lee J.H., Jo Y.H., Kim K., Lee J.H., Rim K.P., Kwon W.Y., Suh G.J., Rhee J.E. (2013). Effect of N-Acetylcysteine (NAC) on Acute Lung Injury and Acute Kidney Injury in Hemorrhagic Shock. Resuscitation.

[86] Abdelrazik E.A., Hassan H.M.M., Mahmoud Z.A., Yousef A.M., Elsayed E.A. (2022). Renoprotective Effect of N-Acetylcystein and Vitamin E in Bisphenol A-Induced Rat Nephrotoxicity; Modulators of Nrf2/ NF-ΚB and ROS Signaling Pathway. Acta Biomed..

[87] Pedre B., Barayeu U., Ezeriņa D., Dick T.P. (2021). The Mechanism of Action of N-Acetylcysteine (NAC): The Emerging Role of H_2_S and Sulfane Sulfur Species. Pharmacol. Ther..

[88] Kong X., Wang R., Xue Y., Liu X., Zhang H., Chen Y., Fang F., Chang Y. (2010). Sirtuin 3, a New Target of PGC-1α, Plays an Important Role in the Suppression of ROS and Mitochondrial Biogenesis. PLoS ONE.

[89] Peerapanyasut W., Kobroob A., Palee S., Chattipakorn N., Wongmekiat O. (2019). Activation of Sirtuin 3 and Maintenance of Mitochondrial Integrity by N-Acetylcysteine Protects against Bisphenol A-Induced Kidney and Liver Toxicity in Rats. Int. J. Mol. Sci..

[90] Sharma M., Kaur T., Singla S.K. (2015). Protective Effects of N-Acetylcysteine against Hyperoxaluria Induced Mitochondrial Dysfunction in Male Wistar Rats. Mol. Cell. Biochem..

[91] Zhao W., Zhang L., Chen R., Lu H., Sui M., Zhu Y., Zeng L. (2018). SIRT3 Protects against Acute Kidney Injury via AMPK/MTOR-Regulated Autophagy. Front. Physiol..

[92] Nam B.Y., Jhee J.H., Park J.T.J., Kim S., Kim G., Park J.T.J., Yoo T.H., Kang S.W., Yu J.W., Han S.H. (2022). PGC-1α Inhibits the NLRP3 Inflammasome via Preserving Mitochondrial Viability to Protect Kidney Fibrosis. Cell Death Dis..

[93] Gao Q., Zhu H. (2019). The Overexpression of Sirtuin1 (SIRT1) Alleviated Lipopolysaccharide (LPS)-Induced Acute Kidney Injury (AKI) via Inhibiting the Activation of Nucleotide-Binding Oligomerization Domain-like Receptors (NLR) Family Pyrin Domain Containing 3 (NLRP3) Inflammas. Med. Sci. Monit..

[94] Li Q., Liao J., Chen W., Zhang K., Li H., Ma F., Zhang H., Han Q., Guo J., Li Y. (2022). NAC Alleviative Ferroptosis in Diabetic Nephropathy via Maintaining Mitochondrial Redox Homeostasis through Activating SIRT3-SOD2/Gpx4 Pathway. Free Radic. Biol. Med..

[95] Hostetter T.H., Olson J.L., Renke H.G., Venkatachalam M.A., Brenner B.M. (2001). Hyperfiltration in Remnant Nephrons: A Potentially Adverse Response to Renal Ablation. J. Am. Soc. Nephrol..

[96] Brenner B.M. (1985). Nephron Adaptation to Renal Injury or Ablation. Am. J. Physiol..

[97] Taal M.W., Brenner B.M., Skorecki K., Chertow G.M., Marsden P.A., Taal M.W., Yu A.S.L. (2012). Adaptation to Nephron Loss and Mechanisms of Progression in Chronic Kidney Disease. Brenner & Rector’s The Kidney.

[98] Sinuani I., Averbukh Z., Gitelman I., Rapoport M.J., Sandbank J., Albeck M., Sredni B., Weissgarten J. (2006). Mesangial Cells Initiate Compensatory Renal Tubular Hypertrophy via IL-10-Induced TGF-Beta Secretion: Effect of the Immunomodulator AS101 on This Process. Am. J. Physiol. Renal Physiol..

[99] Hauser P., Kainz A., Perco P., Bergmeister H., Mitterbauer C., Schwarz C., Regele H.M., Mayer B., Meyer T.W., Oberbauer R. (2005). Transcriptional Response in the Unaffected Kidney after Contralateral Hydronephrosis or Nephrectomy. Kidney Int..

[100] Wolf G., Ziyadeh F.N. (1999). Molecular Mechanisms of Diabetic Renal Hypertrophy. Kidney Int..

[101] Ceja-Galicia Z.A., García-Arroyo F.E., Aparicio-Trejo O.E., El-Hafidi M., Gonzaga-Sánchez G., León-Contreras J.C., Hernández-Pando R., Guevara-Cruz M., Tovar A.R., Rojas-Morales P. (2022). Therapeutic Effect of Curcumin on 5/6Nx Hypertriglyceridemia: Association with the Improvement of Renal Mitochondrial β-Oxidation and Lipid Metabolism in Kidney and Liver. Antioxidants.

[102] He J., Wang Y., Sun S., Yu M., Wang C., Pei X., Zhu B., Wu J., Zhao W. (2012). Bone Marrow Stem Cells-Derived Microvesicles Protect against Renal Injury in the Mouse Remnant Kidney Model. Nephrology.

[103] Wu M., Xia W., Jin Q., Zhou A., Wang Q., Li S., Huang S., Zhang A., Zhang Y., Li Y. (2021). Gasdermin E Deletion Attenuates Ureteral Obstruction- and 5/6 Nephrectomy-Induced Renal Fibrosis and Kidney Dysfunction. Front. Cell Dev. Biol..

[104] Tamaki M., Miyashita K., Wakino S., Mitsuishi M., Hayashi K., Itoh H. (2014). Chronic Kidney Disease Reduces Muscle Mitochondria and Exercise Endurance and Its Exacerbation by Dietary Protein through Inactivation of Pyruvate Dehydrogenase. Kidney Int..

[105] Askari H., Seifi B., Kadkhodaee M. (2016). Evaluation of Renal-Hepatic Functional Indices and Blood Pressure Based on the Progress of Time in a Rat Model of Chronic Kidney Disease. Nephrourol. Mon..

[106] Oppenheimer H., Gabay O., Meir H., Haze A., Kandel L., Liebergall M., Gagarina V., Lee E.J., Dvir-Ginzberg M. (2012). 75-Kd Sirtuin 1 Blocks Tumor Necrosis Factor α-Mediated Apoptosis in Human Osteoarthritic Chondrocytes. Arthritis Rheum..

