# Supplementary material for: Early Administration of N-Acetylcysteine Provides Renal and Cardiac Mitochondrial and Redox Protection, Preventing the Development of Cardio-Renal Syndrome Type IV Induced by 5/6NX
Source: Antioxidants (Basel). 2025 Oct 16;14(10):1241. doi: 10.3390/antiox14101241 (PMC12561179; doi:10.3390/antiox14101241)
Supplement: Supplementary file 1 [file antioxidants-14-01241-s001.zip › antioxidants-3839327-Supplementary.pdf]

Supplementary material

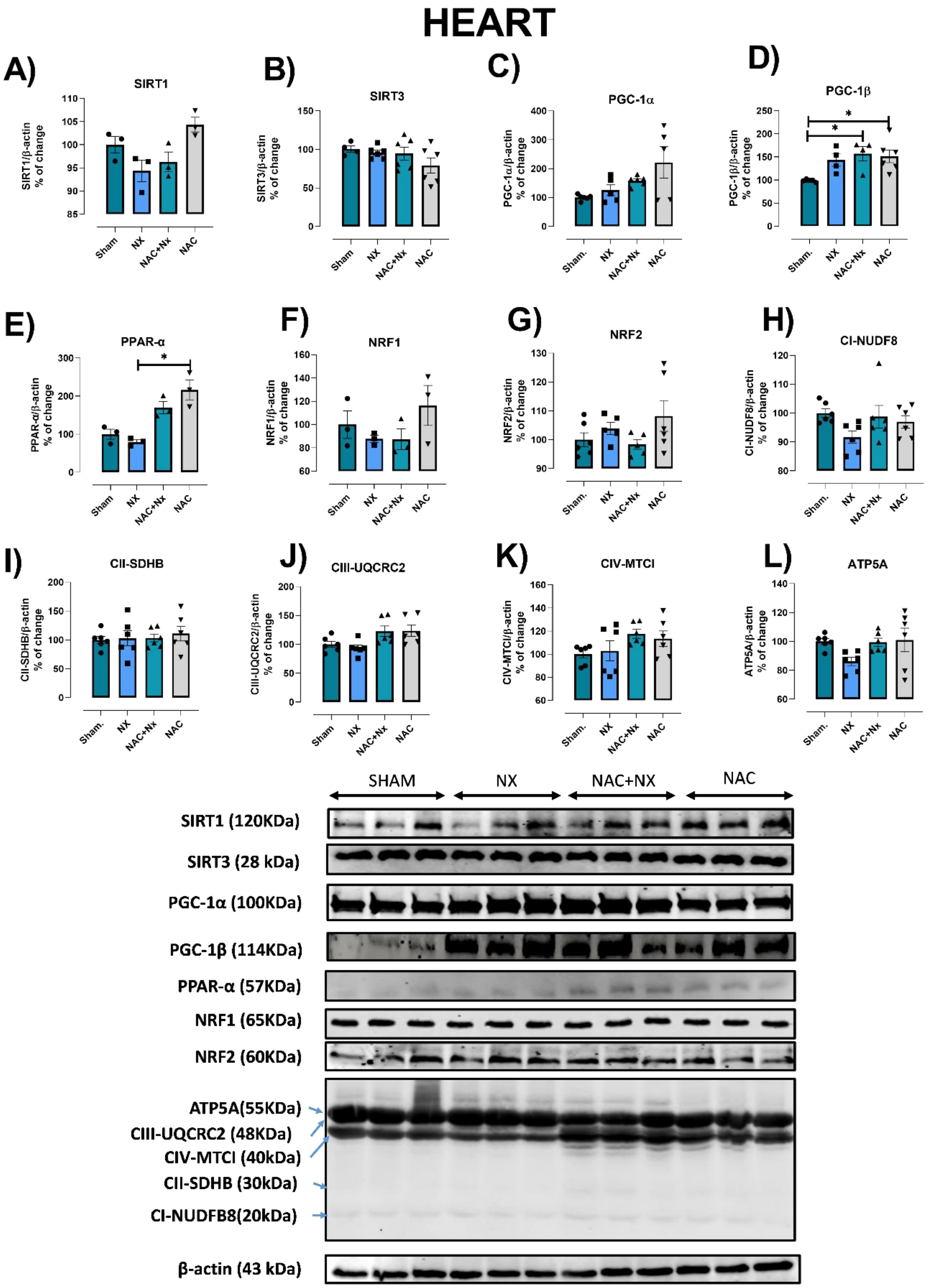

Supplementary Figure S1. Evaluation in heart of mitochondrial biogenesis factors by Western blot and its densitometry at 10 days after surgery: (**A**) SIRT1, (**B**) SIRT3, (**C**) PGC-1α, (**D**) PGC-1β, (**E**) PPARα, (**F**) NRF1, (**G**) NRF2, (**H**) CI-NDUFB8, (**I**) CII-SDHB, (**J**) CIII-UQCRC2, (**K**) CIV-MTCO1, (**L**) ATP5A. B-actin of the corresponding membranes was used as a protein loading control. Data are the mean ± SEM, n = 3-6. * $p < 0.05$. Kruskal-Wallis test followed by a Dunn's post hoc test NX= 5/6 nephrectomy, NAC = N-acetylcysteine. CI-NDUFB8= reduced form of the nicotinamide adenine dinucleotide ubiquinone oxidoreductase subunit B8; CII-SDHB= succinate dehydrogenase complex iron-sulfur subunit B, CIII-UQCRC2= ubiquinol-cytochrome c reductase core protein 2, CIV-MTCO1= cytochrome c oxidase subunit I, ATP5A= adenine triphosphate (ATP) synthase subunit α, NRF1= nuclear respiratory factor 1, NRF2= nuclear respiratory factor 2, NX= 5/6 nephrectomy, NAC = N-acetyl-cysteine, PGC-1α= peroxisome proliferator-activated receptor gamma coactivator 1-alpha, PGC-1β= peroxisome proliferator-activated receptor gamma coactivator 1-beta, PPAR-α= peroxisome proliferator-activated receptor alpha, SIRT1= sirtuin 1, SIRT3= sirtuin

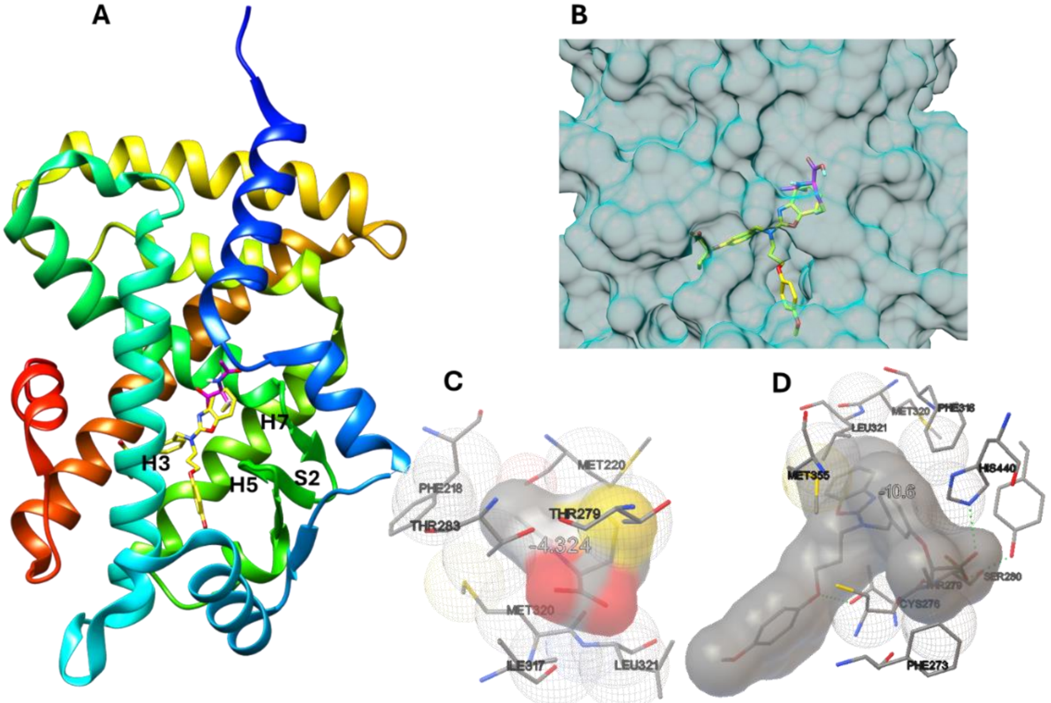

Supplementary Figure S2. (**A**) Ribbons representation of the complex PPAR-α (PDB code: 6KB4) with pemafibrate and NAC^{-}, yellow and magenta stick respectively. (**B**) Hydrophobic pocket where the two ligands interact, pemafibrate and NAC^{-} both compounds share a region of interaction in the H3, H5, H7, and S2. (**C**) Specific interactions PPAR-α−NAC^{-}, all corresponding to Van der Waals forces. (**D**) Specific interactions PPAR-α−pemafibrate, due to greater molecular area, the number of interactions increases, standing out the hydrogen bonds with Ser280, Tyr314, His440, and Tyr464. NAC = N-acetylcysteine.

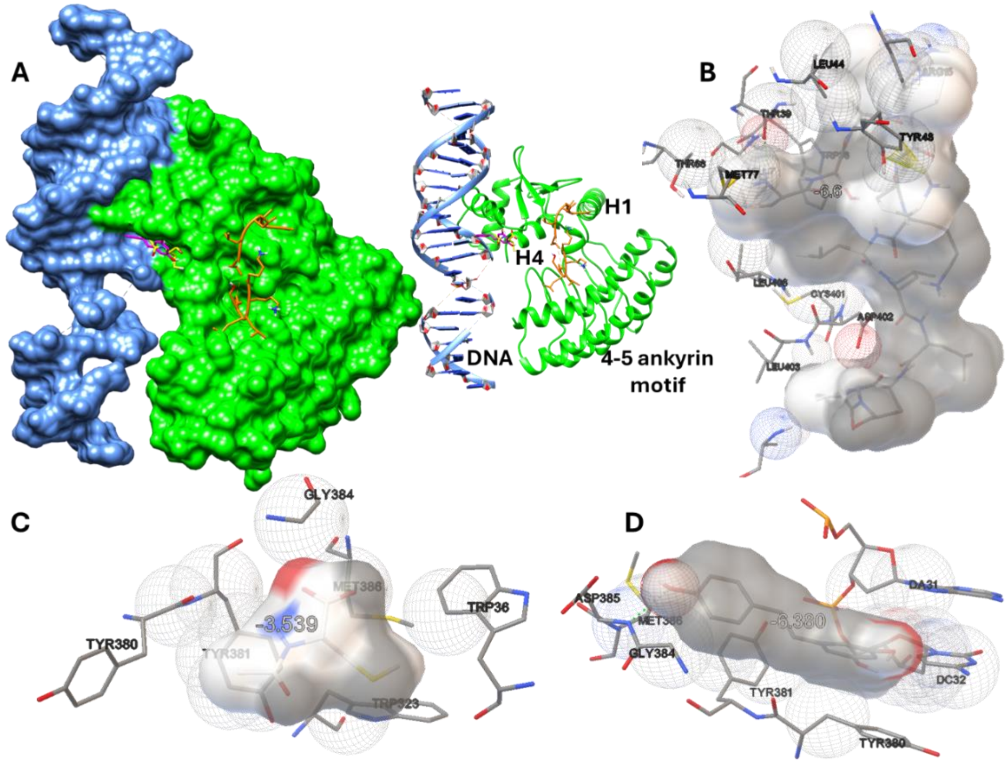

Supplementary Figure S3. (**A)** Ribbons representation of the complex NRF2 (PDB code: 1AWC) with PGC-1α, resveratrol and NAC⁻; orange, yellow and magenta stick respectively. (**B**) Hydrophobic pocket where PGC-1α interact with NRF2 and the specific interactions presented. (**C**) Specific interactions NRF2−NAC⁻, all corresponding to Van der Waals forces. (**D**) Specific interactions NRF2−resveratrol, due to the larger molecular area, the number of interactions increases. NAC⁻ and resveratrol share a region of interaction in the GABPα and DNA interface. NAC = N-acetylcysteine.

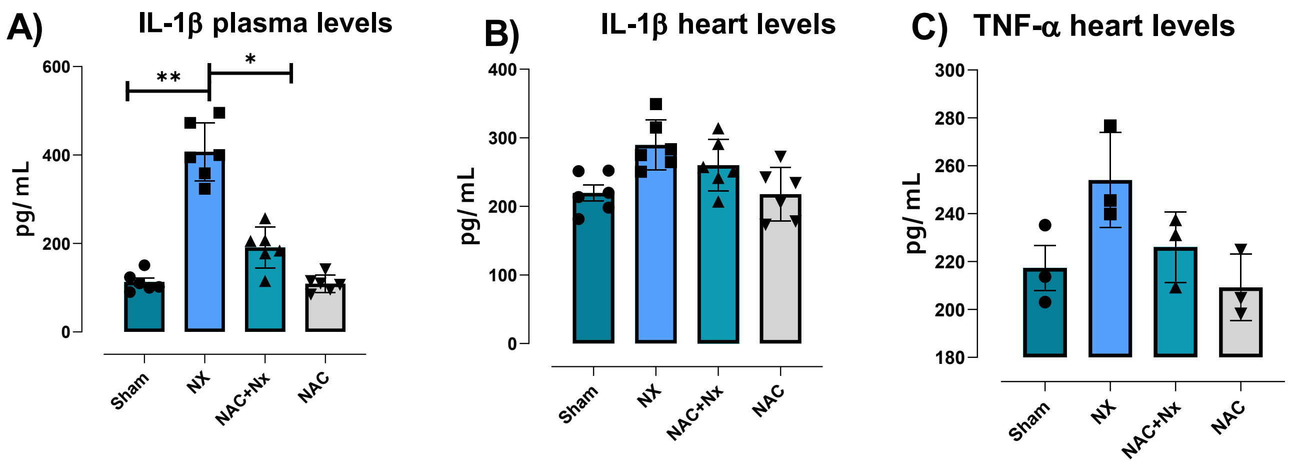

Supplementary Figure S4. Evaluation in plasma (**A**) and heart (**B**) of proinflammatory cytokine interleukin one beta (IL-1β) and (**C**) necrosis tumor factor alpha (TNF-α) in heart by ELISA kits. Data are mean ± SEM, n =3- 6. *p<0.05, ** p < 0.01. Kruskal-Wallis test followed by a Dunn's post hoc test NX= 5/6 nephrectomy, NAC = N-acetylcysteine.

Supplementary Table S1. List of probes, antibodies, reagents and consumables used, as well as their catalog numbers and manufacturing companies

Name	Catalogue number	Manufacturer	Dilution of the antibody (if is applicable)
Adenosine 5′-diphosphate sodium salt (ADP)	A2754	Sigma-Aldrich	-
Ampliflu™ Red	90101	Sigma-Aldrich	-
Antimycin A	A8674	Sigma-Aldrich	-
Anti-β-Actin antibody	sc-47778	Santa Cruz	1:10000
Anti-PGC1 alpha antibody	ab191838	Abcam	1:2500
Anti-IL6 antibody	GTX110527	GeneTex	1:3000
Anti-IL-1 beta/IL1B antibody	sc-515598	Santa Cruz	1:2000
Anti-Cardiac Troponin T antibody	GTX134489	GeneTex	1:5000
Anti-GADPH antibody	sc-365062	Santa Cruz	1:10000
Anti-PGC1 alpha antibody	sc-518038	Santa Cruz	1:5000
Anti-PGC1 beta antibody	sc-517279	Santa Cruz	1:5000
Anti-VDAC antibody	4866	Cell Signaling	1:2500
Anti-TNF-alpha antibody	sc-52746	Santa Cruz	1:2500

Anti-Sirt3 antibody	sc-99143	Santa Cruz	1:2000
Anti-NRF1 antibody	sc-33771	Santa Cruz	1:5000
Anti-NRF2/GABP-α antibody	sc-28312	Santa Cruz	1:5000
Anti-Sirt1 antibody	8469	Cell Signaling	1:2500
Anti- PPAR alpha Antibody	sc-398394	Santa Cruz	1:2500
Anti-ANT	ab220408	Abcam	1:5000
Anti-BNP	sc-18818	Santa Cruz	1:5000
Fat-free bovine serum albumin (BSA)	A6003	Sigma-Aldrich	-
B-mercaptoethanol	M3148	Sigma-aldrich	-
Bromophenol blue	114391	Sigma-aldrich	-
1-Chloro-2,4-dinitrobenzene (CDNB)	237329	Sigma-Aldrich	-
Carbonyl cyanide m-chlorophenylhydrazone (CCCP)	C2759	Sigma-Aldrich	-
Cytochrome *c* from equine heart	C7752	Sigma-Aldrich	-
D-mannitol		Sigma-Aldrich	-
Decylubiquinone (DUB)	D7911		-
2,6-dichlorophenolindophenol sodium salt hydrate (DCPIP),	M9647	Sigma-Aldrich	-
5,5′-dithio-bis-(2-nitrobenzoic acid) (DTNB)	D218200	Sigma-Aldrich	-
Dithiothreitol (DTT)	43816	Sigma-Aldrich	-
Donkey Anti-Goat IgG Secondary Antibody IRDye® 680RD	926-68074	LCR-R	1:10000
Glutathione (GSH)	G6013	Sigma-Aldrich	-
Glutathione disulfide (GSSG)	G4501	Sigma-Aldrich	-
GSH peroxidase (Gpx)	G6137	Sigma-Aldrich	-
Ethylene glycol-bis(2-aminoethyl ether)-N,N,N′,N′-tetraacetic acid (EGTA)	E3889	Sigma-Aldrich	-
Glucose-6-phosphate dehydrogenase (G6PDH)	G7877	Sigma-Aldrich	-
Glutathione reductase (GR)	G3664	Sigma-Aldrich	-
Glutamic acid	G1251	Sigma-aldrich	-
Glutaraldehyde	G7651	Sigma-aldrich	-
Goat Anti-Rabbit IgG Secondary Antibody IRDye® 680RD	926-68071	LCR-R	1:10000
Goat Anti-Mouse IgG I Secondary Antibody RDye® 680RD	926-68070	LCR-R	1:10000
Goat anti-Rabbit IgG Secondary Antibody IRDye® 800CW	926-32211	LCR-R	1:20000
Goat anti-Mouse IgG Secondary Antibody IRDye® 800CW	925-32210	LCR-R	1:20000

Hexokinase	H6380	Sigma-Aldrich	-
4-(2-hydroxyethyl)-1-piperazineethanesulfonic acid (HEPES)	H4034	Sigma-Aldrich	-
Horseradish peroxidase (HRP)	77332	Sigma-Aldrich	-
Lactobionic acid	153516	Sigma-Aldrich	-
Manganese (II) chloride ($MgCl_2$)	13220	Sigma-Aldrich	-
Malic acid	240176	Sigma-aldrich	-
NAC	A0737	Sigma-Aldrich	-
β-Nicotinamide adenine dinucleotide phosphate reduced (NADPH),	N7505	Sigma-Aldrich	-
Oxidized ($NADP^+$)	N5755	Sigma-Aldrich	-
OxPhos Rodent WB Antibody Cocktail	45-8099	Invitrogen/ ThermoFisher	1:5000
B-Nicotinamide adenine dinucleotide reduced (NADH)	N8129	Sigma-Aldrich	-
Oxidized Nicotinamide adenine dinucleotide (NAD^+)	N3886	Sigma-Aldrich	-
Nitro blue tetrazolium (NBT),	N5514	Sigma-Aldrich	-
Potassium cyanide (KCN),	207810	Sigma-Aldrich	-
Rotenone	45656	Sigma-aldrich	-
Safranin O	S2255	Sigma-Aldrich	-
Sodium azide	S2002	Sigma-aldrich	-
Sodium succinate dibasic	S2378	Sigma-aldrich	-
Sodium phosphate dibasic	S9763	Sigma-Aldrich	-
Sodium phosphate monobasic	S9638	Sigma-Aldrich	-
Sodium glutamate	G1251	Sigma-aldrich	-
Sodium chloride	S5886	Sigma-Aldrich	-
Sodium fluoride	201154	Sigma-Aldrich	-
Sodium orthovanadate (Na_3VO_4)	S6508	Sigma-Aldrich	-
Sodium malate	M9138	Sigma-aldrich	-
Sodium deoxycholate	S1827	Sigma-aldrich	-
Sodium dodecyl sulfate (SDS)	L4509	Sigma-Aldrich	-
Paraformaldehyde	158127	Sigma-aldrich	-
Sucrose	S9378	Sigma-aldrich	-
Taurine	T0625	Sigma-aldrich	-
Tetramethyl-p-phenylenediamine (TMPD)	T7394	Sigma-Aldrich	-
Trizma base	93352	Sigma-Aldrich	-
Trizma-hydrochloride	T5941	Sigma-Aldrich	-
Triton X-100	T9284	Sigma-Aldrich	-
Thiamine pyrophosphate	C8754	Sigma-aldrich	-
Tween	STS0200	Sigma-aldrich	-

Osmium tetroxide	201030	Sigma-aldrich	-
NADP/NADPH quantitation kit	MAK037	Sigma-Aldrich	-
Creatine kinase (CK) activity assay kit	MAK116	Sigma-Aldrich	-
Blood urea nitrogen kit	1001323	Spinreact	-
Plasma creatinine kit	1001110	Spinreact	-
Sodium pentobarbital/ SedalphorteMR	Q-7503-003	Salud y Bienestar Animal S.A. de C.V	-
Sodium bicarbonate	02-003-990	JT Baker	-
H_2O_2	15587984	JT Baker	-
Ethyl alcohol	10375842	JT Baker	-
Ethylenediaminetetraacetic acid disodium salt dihydrate (EDTA)	10213570	JT Baker	-
Potassium hydroxide	10192830	JT Baker	-
Protease inhibitor cocktail	11697498001	Roche Applied Science	-
Phosphoenolpyruvate	10005185103	Roche Applied Science	-
Rat IL-1 beta ELISA Kit	Ab100768	Abcam	-
Rat TNF alpha ELISA Kit	Ab100785	Abcam	
